# Understanding How the Design and Implementation of Online Consultations Affect Primary Care Quality: Systematic Review of Evidence With Recommendations for Designers, Providers, and Researchers

**DOI:** 10.2196/37436

**Published:** 2022-10-24

**Authors:** Sarah Darley, Tessa Coulson, Niels Peek, Susan Moschogianis, Sabine N van der Veer, David C Wong, Benjamin C Brown

**Affiliations:** 1 Division of Population Health, Health Services Research and Primary Care School of Health Sciences The University of Manchester Manchester United Kingdom; 2 National Health Service Salford Clinical Commissioning Group Salford United Kingdom; 3 Centre for Health Informatics Division of Informatics, Imaging and Data Science, Manchester Academic Health Science Centre The University of Manchester Manchester United Kingdom; 4 National Institute for Health and Care Research Greater Manchester Patient Safety Translational Research Centre School of Health Sciences The University of Manchester Manchester United Kingdom; 5 National Institute for Health and Care Research Applied Research Collaboration Greater Manchester Manchester United Kingdom; 6 Department of Computer Science The University of Manchester Manchester United Kingdom

**Keywords:** general practice, systematic review, remote consultation, OC, triage, primary health care, care provider, health care professional, workforce, telemedicine, COVID-19, pandemic, primary care, health outcome, patient care

## Abstract

**Background:**

Online consultations (OCs) allow patients to contact their care providers on the web. Worldwide, OCs have been rolled out in primary care rapidly owing to policy initiatives and COVID-19. There is a lack of evidence regarding how OC design and implementation influence care quality.

**Objective:**

We aimed to synthesize research on the impacts of OCs on primary care quality, and how these are influenced by system design and implementation.

**Methods:**

We searched databases from January 2010 to February 2022. We included quantitative and qualitative studies of real-world OC use in primary care. Quantitative data were transformed into qualitative themes. We used thematic synthesis informed by the Institute of Medicine domains of health care quality, and framework analysis informed by the nonadoption, abandonment, scale-up, spread, and sustainability framework. Strength of evidence was judged using the GRADE-CERQual approach.

**Results:**

We synthesized 63 studies from 9 countries covering 31 OC systems, 14 (22%) of which used artificial intelligence; 41% (26/63) of studies were published from 2020 onward, and 17% (11/63) were published after the COVID-19 pandemic. There was no quantitative evidence for negative impacts of OCs on patient safety, and qualitative studies suggested varied perceptions of their safety. Some participants believed OCs improved safety, particularly when patients could describe their queries using free text. Staff workload decreased when sufficient resources were allocated to implement OCs and patients used them for simple problems or could describe their queries using free text. Staff workload increased when OCs were not integrated with other software or organizational workflows and patients used them for complex queries. OC systems that required patients to describe their queries using multiple-choice questionnaires increased workload for patients and staff. Health costs decreased when patients used OCs for simple queries and increased when patients used them for complex queries. Patients using OCs were more likely to be female, younger, and native speakers, with higher socioeconomic status. OCs increased primary care access for patients with mental health conditions, verbal communication difficulties, and barriers to attending in-person appointments. Access also increased by providing a timely response to patients’ queries. Patient satisfaction increased when using OCs owing to better primary care access, although it decreased when using multiple-choice questionnaire formats.

**Conclusions:**

This is the first theoretically informed synthesis of research on OCs in primary care and includes studies conducted during the COVID-19 pandemic. It contributes new knowledge that, in addition to having positive impacts on care quality such as increased access, OCs also have negative impacts such as increased workload. Negative impacts can be mitigated through appropriate OC system design (eg, free text format), incorporation of advanced technologies (eg, artificial intelligence), and integration into technical infrastructure (eg, software) and organizational workflows (eg, timely responses).

**Trial Registration:**

PROSPERO CRD42020191802; https://tinyurl.com/2p84ezjy

## Introduction

### Background

Online consultation (OC) systems allow patients to contact their health care provider over the internet to ask health-related questions and report symptoms [1]. Their query may then be resolved with a written response, telephone call, video consultation, or in-person visit. Many terms are used to describe this type of technology, including *e-consultation*, *e-visit*, and *online triage* (Multimedia Appendix 1 [2-28])—in this review, we refer to them all as *online consultations*. We distinguish OCs from “symptom checkers” [29] and other self-service systems that typically do not directly facilitate communication with a human health care provider and from patient portals [30], which may include generic email or secure messaging functionalities.

OCs are considered by policy makers in many countries as a way to address the increasing workload and decreasing workforce capacity in primary care [31-36] while still meeting patient expectations and improving access [37]. However, they have the potential to exacerbate health inequities [38,39] and increase inappropriate antibiotic prescriptions [40]. Furthermore, there are widely recognized challenges in initiating and sustaining the adoption of new technologies in primary care [41].

Although symptom checkers [29,42] and patient portals [30,43,44] have been well studied, only a small number of evidence syntheses directly relevant to OCs have been published: a systematic review of 57 articles on delivering “e-consultation” in primary care largely focused on generic stand-alone applications such as email and video (n=39/57, 68%) [45]; a scoping review of “online triage tools” included 13 papers, 4 of which (31%) were nonempirical (eg, opinion pieces) [46]; and a review of 17 studies of “intelligent online triage tools” focused only on those that used “artificial intelligence” (AI) [47].

Since these syntheses were conducted, OCs have gained wider traction in clinical practice worldwide—they have been indispensable in helping manage patients remotely to minimize the spread of COVID-19 [48,49], and English primary care providers have been mandated to offer OCs for all patients since April 2020 [50]. Moreover, OC system product design has progressed significantly to become more specialized and technologically advanced [51], with several more empirical research studies published on their use [2-11,52-64].

Given this rapid scale-up and increase in the diversity and complexity of OCs, further insight is needed into their impact on health care quality. Previous reviews have not reported the design or implementation details of the OCs they studied [45-47] despite their importance in understanding the causal mechanisms of how they affect care outcomes [65]. The aim of this study was to systematically review and synthesize the empirical quantitative and qualitative literature in a theoretically informed way to address this knowledge gap.

### Objectives

Informed by existing theories, the aim of this study was to synthesize quantitative and qualitative research on (1) the impacts of OCs on primary care quality and (2) how these are influenced by OC system design and implementation.

## Methods

### Study Design

We consider OCs as complex interventions and, therefore, synthesized both quantitative and qualitative evidence to understand their impacts in specific contexts [66]. We did not perform a meta-analysis because of the heterogeneous and nonrandomized nature of the included studies [67]. We followed the PRISMA (Preferred Reporting Items for Systematic Reviews and Meta-Analyses) statement [68].

### Registration and Protocol

The study protocol was registered with PROSPERO (CRD42020191802) [69]. The original title was amended to be less general and more specific to the objectives of the review, and the objectives were amended to focus on care quality.

### Inclusion Criteria

Papers that met the following criteria were included: empirical studies using quantitative or qualitative methods to examine the real-world use of OCs in primary care in any country, written in English, and published in 2010 or later. We excluded news articles, opinion pieces, literature reviews, non–English-language articles, and literature published before 2010.

We defined OCs as digital interventions that allow patients to contact their primary care provider by inputting “queries” into health care–specific web-based forms [1]. We included symptom checkers and similar self-service systems [54] if at least one of their outcomes directly facilitated contact with a primary care health professional. We included patient portals if they had a secure messaging functionality that used health care–specific forms [54]. We excluded stand-alone generic communication technologies such as email or videoconferencing software.

### Search Strategy

We searched the Ovid MEDLINE, EMBASE, Web of Science, and Scopus databases during July 2020 (Multimedia Appendix 2 [12,53,56,58-60,63,70-73]). Our search strategy was developed from scoping searches of the literature and drew on search strategies used in related literature reviews [45,46]. We searched the National Technical Information Service, the Health Management Information Consortium, and Zetoc to find relevant gray literature, conference proceedings, and theses. We found further literature through citation mapping and in the reference lists of the included papers, searching during August 2020 and September 2020. SD and TC independently screened titles and abstracts and then full papers for eligibility, resolving differences through discussion at each stage. All literature searches were rerun by SD between November 2021 and February 2022.

### Data Extraction and Quality Appraisal

We extracted data from the included papers as verbatim text, capturing study characteristics (eg, research design and study setting) and key findings relevant to our research objectives based on the nonadoption, abandonment, scale-up, spread, and sustainability (NASSS) framework [74] (Multimedia Appendix 3). We used the NASSS to capture “a rich, contextualised narrative of technology-supported change efforts and the numerous interacting influences that help explain its successes, failures, and unexpected events” [75]. The methodological quality of the studies was assessed using the Mixed Methods Appraisal Tool (MMAT), which is designed for qualitative, quantitative, and mixed methods studies [76]. We scored each paper using recommended quintile percentages as cutoffs and considered any paper scoring at least 60% as of “good” quality [77]. SD and TC extracted data from 10 papers independently, which confirmed high interrater agreement. Following this, SD extracted data from the remaining papers, which were checked by TC.

### Data Synthesis

The data were imported into NVivo (version 12; QSR International) [78] for synthesis. To integrate both quantitative and qualitative data, during data synthesis, quantitative data were transformed into qualitative themes (“qualitising”) [79].

For objective 1, we considered “impacts of OCs on primary care quality” as consequences of using OCs that could relate to patients, primary care staff, or the wider system [65]. We used thematic synthesis [80], which involved SD and TC coding the text from the data extraction forms independently line by line, developing higher-level themes through regular discussion [80]. Impacts on care quality were synthesized inductively, with emerging themes mapped to the six Institute of Medicine domains of health care quality [81]: safe (avoiding harm to patients from care that is intended to help), effective (providing care based on scientific knowledge to produce better clinical outcomes), patient-centered (care that is respectful and responsive), timely (reducing waits and delays for those who receive and give care), efficient (avoiding waste), and equitable (care that does not vary in quality because of personal characteristics) [81]. Our emergent findings suggested that OCs had both positive and negative impacts and, therefore, theme descriptions were edited to be neutral (eg, safe→safety and efficient→efficiency).

For objective 2, we considered OC “design” as material properties of an OC, such as features and functionality [74], and “implementation” as the way an OC was introduced and used in a particular context [65]. As a design feature, we considered AI as the ability of machines to “mimic human intelligence as characterized by behaviors such as cognitive ability, memory, learning, and decision making” [82]. We synthesized the extracted data using framework analysis [83], which involved SD and TC reading and rereading each data extraction form and then coding them line by line independently—both deductively by using domains from the NASSS framework [74] for high-level themes and inductively by identifying additional subthemes. Through discussion, SD and TC summarized the findings into five high-level themes: condition complexity (health condition and the illness the OC is used for), technology (material properties of the OC and required knowledge for use), adopters (staff, patients, and carers expected to use the OC), organization (extent of work needed for implementation of the OC, capacity, and readiness), and wider system (policy context) [74]. Two NASSS domains—value proposition (value of the OC to the developer, patients, and health care system) and embedding and adaptation over time (learning and adaptation to changing contexts)—had limited applicability to our findings and were not included in the final synthesis. Informed by realistic evaluation [65], we considered our themes as contextual factors and identified patterns of explanations for how each led to the impacts on care quality from objective 1 (ie, “causal mechanisms”). Where appropriate, we considered the levels of OC adoption as a mechanism for how they affected care quality [65]. We used visual mapping to identify commonalities and discordances in causal mechanisms—first within individual papers and then across papers [83]. Where there were discordances, we explored potential explanations where possible (eg, related to the study setting).

The strength and quality of our findings for objectives 1 and 2 were assessed using the Grading of Recommendations Assessment, Development, and Evaluation-Confidence in Evidence from Reviews of Qualitative Research method [84]. This accounts for the methodological limitations of the contributing papers (according to MMAT assessments), relevance to the review question, coherence of the finding, and adequacy of its supporting data [84]. Confidence in each finding was designated as high, moderate, low, or very low. At each stage of the analysis, the findings were discussed and agreed upon with the wider study team. BCB reviewed all coded verbatim excerpts from the papers included in the final synthesis.

## Results

### Descriptive Summary

**Figure 1:**
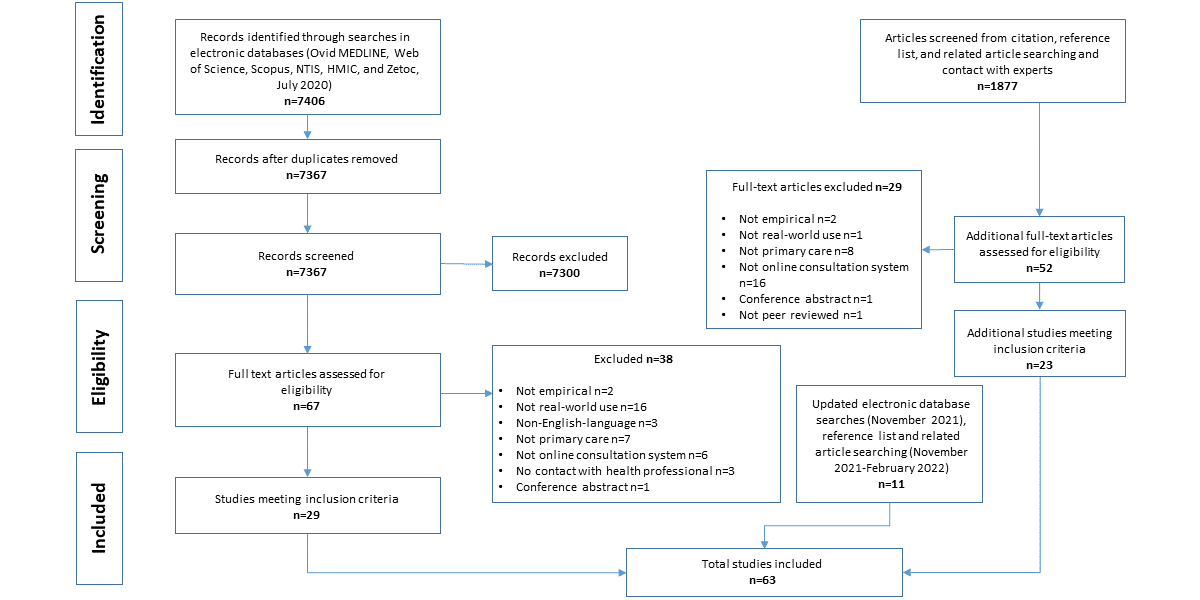
Flowchart of the study selection process. HMIC: Health Management Information Consortium; NTIS: National Technical Information Service.

We synthesized 63 papers (Figure 1), including 52 (83%) journal papers [53], 7 (11%) evaluation reports [85], 3 (5%) conference papers [12], and 1 (2%) master’s degree thesis [13]. The studies were quantitative (33/63, 52%), qualitative (12/63, 19%), and mixed methods (18/63, 29%) and analyzed data from patients (16/63, 25% qualitative studies and 18/63, 29% quantitative studies), staff (22/63, 35% qualitative studies and 9/63, 14% quantitative studies), and clinical systems (33/63, 52% quantitative studies). All were set in one of 9 high-income countries, with most coming from the United States (21/63, 33%) and the United Kingdom (20/63, 32%; Multimedia Appendix 4 [2-27,52-64,70-73,77,85-104]). In all, 41% (26/63) of the studies were published in 2020 or later, and 17% (11/63) were conducted after the start of the COVID-19 pandemic. Examples of excluded studies are those that focused on stand-alone video consultations [105], involved communication between physicians and not patients [106], and were not based on primary care [107].

In all, 83% (52/63) of the studies reported levels of OC adoption by patients and staff, of which 62% (32/52; 32/63, 51% of all studies) were described as “low” by the study authors [86]. OCs were adopted at a high rate in 63% (33/52; 33/63, 52% of all) of the studies [87], including high rates of adoption by certain patient groups even when overall OC adoption in the study was low [14].

The included papers described 31 OC systems summarized in Table 1 and detailed in Multimedia Appendix 5 [2-27,52-64,70-73,85-104]. In 25% (16/63) of the papers, the OC system was described sufficiently to meet our inclusion criteria but not in enough detail to determine specific design features. Of the 31 OCs described, most (23/31, 74%) offered two-way written communication between patients and staff [88], with a few (4/31, 13%) also offering communication by video [52]. In all, 13% (4/31) did not provide functionalities for staff to reply to patients via the system (ie, one-way communication only [14]). In total, 35% (11/31) required patients to describe their queries solely via multiple-choice questionnaires (MCQs) [89] compared with 13% (4/31) that solely required patients to describe their queries using unstructured free text [56]. In all, 42% (13/31) had a hybrid approach of primarily using MCQs with the option for patients to enter additional free text [90]. No free text OCs offered optional MCQs. In all, 26% (8/31) of the OC systems were integrated with the electronic health record (EHR) [58], and 3% (1/31) allowed patients to schedule telephone or in-person appointments with health care professionals themselves [54].

In total, 54% (13/24) of MCQ-based OC systems exhibited three types of AI: (1) adapting questions they asked patients as they submitted their query in response to previous answers given (10/31, 32%) [91]; (2) prioritizing patient queries based on clinical urgency (4/31, 13%) [54]; and (3) signposting patients to an appropriate care provider based on their query, such as self-care, primary care, or emergency department (3/31, 10%) [8]. These were mostly powered by preprogrammed logic and “algorithms” (10/31, 32%) [54], with the exact AI methodology unclear in the remainder (3/31, 10%) [15].

The methodological quality of most studies (42/63, 67%) was “good” (ie, ≥60% according to the MMAT [77]; Multimedia Appendix 6 [2-27,52-64,70-73,76,85-104]). Common limitations included a lack of detail on whether the OC was administered as intended [92] and small sample sizes [3].

**Table 1:** Online consultation (OC) system features (N=31).

OC system feature and subcategory			Studies, n (%)^a^
**Communication mode**			
	Two-way written communication between staff and patients	23 (74)	
	One-way written communication (staff cannot reply to patients)	4 (13)	
	Videoconferencing	4 (13)	
	Unclear	4 (13)	
**Patient query format**			
	Multiple-choice questionnaires only	11 (35)	
	Unstructured free text only	4 (13)	
	Multiple-choice questionnaires with optional free text	13 (42)	
	Unclear	3 (10)	
**Integration with other software**			
	Electronic health record	8 (26)	
	Appointment scheduling	1 (3)	
	No integration	23 (74)	
**Artificial intelligence function**			
	Adapting questions during query submission	10 (32)	
	Prioritizing patient queries based on clinical urgency	4 (13)	
	Signposting patients to the most appropriate care provider	3 (10)	
	No artificial intelligence	17 (55)	
**Artificial intelligence method**			
	Preprogrammed logic and algorithms	10 (32)	
	Unclear	3 (10)	

^a^Count of OC systems described in detail (n=31). Categories may add up to >31 as OC systems may have more than one feature in a category.

### Synthesis

#### Overview

To maintain readability, we present only moderate- and high-confidence findings and provide only 1 example reference per finding. Tables 2 and 3 provide all the references and specify whether the findings are qualitative or quantitative. Multimedia Appendix 7 [13,59,99] and Multimedia Appendix 8 [3, 5, 8-11, 13-17, 19-21, 25, 27, 54, 57, 59, 60, 63, 64, 71, 85, 90, 91, 95, 97, 100, 101] detail the low-confidence findings. Multimedia Appendix 9 [2-27,52,54-61,63,64,67,70-73,85-101] and Multimedia Appendix 10 [3-27, 52, 54-64, 70-73, 85-95, 97, 98, 100, 101] provide exemplar data.

**Table 2:** Impacts of online consultations (OCs) on primary care quality.

Theme	Subtheme
Safety (harm to patients)	Decreased patient safety (qualitative) [2,3,5,7,10,13,17,18,23-25,55,61,63,85,90,94] Description: patient and staff perceptions that OCs worsened patient safety CERQual^a^ rating: high Neutral-increased patient safety (qualitative and quantitative) [3-5,9,11,13,14,16,18,21,54,55,57-59,62,63,70,71,88,89,92,93,95,96] Description: no quantitative evidence of negative impacts on patient safety, with clinician and patient perceptions that OCs improved patient safety CERQual rating: high
Effective (providing care based on scientific knowledge to produce better clinical outcomes)	Reduced antibiotic prescribing rates (quantitative) [15,60,62,97] Description: fewer antibiotics prescribed when using OCs CERQual rating: moderate
Timeliness (reducing waits and delays)	Increased access (qualitative and quantitative) [2-4,6,7,9,13-21,23-25,55-58,62-64,85,90,92,95] Description: easier and more convenient for patients to contact their primary care provider and quicker to communicate with a health professional CERQual rating: high
Efficiency (avoiding waste)	Decreased workload (qualitative and quantitative) [3-5,9,11,13-21,23,54-58,60,61,63,64,70,71,85,89,90,92-95] Description: less work for staff and patients to provide and receive care, respectively CERQual rating: high Increased workload (qualitative and quantitative) [3-5,8-10,13-23,25,52,55,56,58,64,85-87,92,93,98] Description: more work for staff and patients to provide and receive care, respectively CERQual rating: high Decreased costs (qualitative and quantitative) [5,15-18,21,23,56,57,60,61,63,70,85,89,92,95,96,99,100] Description: lower costs for the health care system and patients to provide and receive care, respectively CERQual rating: high Increased costs (qualitative and quantitative) [5,16-19,22,23,63,87] Description: higher costs for the health care system CERQual rating: high
Equitable (variation because of personal characteristics)	Decreased equity (qualitative and quantitative) [7,8,12-27,52,57,59,60,63,64,70-73,85,87-92,94,95,97,98,100,101] Description: OC use variation based on patient characteristics CERQual rating: high Increased equity (qualitative) [7,9,14-20,23,24,27,57,63,64,85,87,90,91] Description: OCs helped patients who had previously struggled because of their personal characteristics communicate with their primary care providers CERQual rating: high
Patient-centeredness (care that is respectful and responsive)	Decreased patient satisfaction (qualitative) [9,11,14,15,18,21,23-25,57,64,85,90] Description: negative patient experiences of using OCs CERQual rating: high Increased patient satisfaction (qualitative and quantitative) [2,5-7,9,11,13-21,23-25,56,57,63,64,71,85,89,90,92-94,96,99] Description: positive patient experiences of using OCs CERQual rating: high

^a^CERQual: Confidence in the Evidence from Reviews of Qualitative Research.

**Table 3:** How the impacts of online consultations (OCs) on primary care quality are influenced by system design and implementation.

Theme and OC design feature or implementation		Impact on care quality (from Table 2)^a^	CERQual^b^ rating and references
**Condition complexity (illness the OC is used for)**			
	Decreased complexity of query Description: patient queries are straightforward and easy to resolve (eg, administrative tasks, minor acute illnesses, and prescription requests)	Efficiency: decreased workload (qualitative and quantitative) Efficiency: decreased health costs (qualitative and quantitative)	CERQual rating: high [5,15-18,23,56,61,64,70,85]
	Increased complexity of query Description: patient queries are not straightforward and easy to resolve (eg, multiple ill-defined symptoms)	Efficiency: increased workload (qualitative) Efficiency: increased health costs (qualitative and quantitative)	CERQual rating: high [5,16-19,22,23]
**Technology (material properties of the OC)**			
	MCQs^c^ Description: patients describe their query by completing questionnaires and selecting their answers from a list	Efficiency: increased workload (qualitative) Patient-centeredness: decreased patient satisfaction (qualitative)	CERQual rating (efficiency): high [5, 9, 10, 14, 17, 18, 20, 21, 23, 25, 55, 64, 86] CERQual rating (patient-centeredness): high [5,9,14,18,20,21,25,64,86]
	Free text input Description: patients describe their query using unstructured text	Efficiency: decreased workload (qualitative and quantitative) Safety: increased patient safety (qualitative)	CERQual rating: high [3,16,21,55,58,93,95]
	Two-way written communication Description: patients and staff are able to send written messages to each other	Efficiency: decreased workload (qualitative and quantitative)	CERQual rating: high [55-58,94,95]
	Nonintegration with core software systems Description: OC systems that operate separately from other software used by the primary care provider	Efficiency: increased workload (qualitative)	CERQual rating: high [3-5,10,13,15,17-21,23,55]
**Adopters (expected users of OCs)**			
	Female sex Description: female patients	High adoption (qualitative and quantitative) Equitable: decreased equity (qualitative and quantitative)	CERQual rating: high [8, 12, 13, 15, 18, 20-23, 27, 52, 57, 60, 70, 72, 73, 87-92, 94, 95, 97, 100, 101]
	Lower age Description: younger patients	High adoption (qualitative and quantitative) Equitable: decreased equity (qualitative and quantitative)	CERQual rating: high [7, 8, 13-15, 18, 19, 21-23, 27, 52, 59, 63, 64, 70, 71, 73, 85, 87-91, 94, 97, 101]
	Native speakers Description: patients who are native speakers of the official language of the country they live in	High adoption (qualitative and quantitative) Equitable: decreased equity (qualitative and quantitative)	CERQual rating: high [18,23,25,57,63,89,98]
	High socioeconomic status Description: patients with higher levels of income and education	High adoption (qualitative and quantitative) Equitable: decreased equity (qualitative and quantitative)	CERQual rating: high [15,18,23-27,57,85,87,90]
	Mental health conditions Description: patients with a mental health diagnosis	Timeliness: increased access (qualitative) Equitable: increased equity (qualitative) Patient-centeredness: increased patient satisfaction (qualitative and quantitative)	CERQual rating: high [9,14,15,18-20,57,64]
	Verbal communication difficulties Description: patients with difficulty communicating verbally (eg, those with hearing loss)	Timeliness: increased access (qualitative) Equitable: increased equity (qualitative) Patient-centeredness: increased patient satisfaction (qualitative and quantitative)	CERQual rating: high [16-19,24,64,90]
	Physical barriers to attending in-person appointments Description: patients cannot easily attend in-person appointments (eg, because of physical disabilities, living far from their primary care provider, work commitments, or care responsibilities)	Timeliness: increased access (qualitative) Equitable: increased equity (qualitative) Patient-centeredness: increased patient satisfaction (qualitative and quantitative)	CERQual rating: high [7,15,18,20,23,63,64,85]
	Preference for traditional consulting methods Description: staff and patients believe in-person consultations are the gold standard	Low adoption (qualitative)	CERQual rating: high [11,18,19,24,26,63,85,93]
**Organization (work needed to implement OCs)**			
	Lack of OC promotion Description: patients are not effectively informed that OCs are available for them to contact their primary care provider	Low adoption (qualitative and quantitative)	CERQual rating: moderate [16,18,24,26,95]
	Timely response Description: primary care providers respond quickly to patients’ OC queries	Patient-centeredness: increased patient satisfaction (qualitative and quantitative) Timeliness: increased access (qualitative)	CERQual rating: high [6,13,20,21,23,25,57]
	Nonintegration with daily workflows Description: primary care provider does not coherently plan OCs into their work processes (eg, by not scheduling clinician time to deal with OCs or not diverting as much incoming patient demand as possible via OCs)	Efficiency: increased workload (qualitative and quantitative)	CERQual rating: high [4,5,13,14,17-20,52,55,85,86,93]
	Sufficient resources allocated to implementing OCs Description: adequate training, staff, and facilities are available to conduct OCs	Efficiency: decreased workload (qualitative)	CERQual rating: high [5,13-15,55,85,86,93]
	Lack of continuity of care Description: OC query is not dealt with by a known or preferred physician	Patient-centeredness: decreased patient satisfaction (qualitative)	CERQual rating: moderate [6,13,15,64,92]
**Wider system (policy context)**			
	Government policy Description: policies mandating OC use (eg, by increasing digital modes of contact with primary care in general or minimizing in-person contact during the COVID-19 pandemic)	High adoption (qualitative and quantitative)	CERQual rating: high [4,15,54,62,63,87]
	Lack of financial support Description: no external funding available to pay ongoing costs of OCs	Low adoption (qualitative and quantitative)	CERQual rating: moderate [5,18,23,63,85]

^a^Includes levels of OC adoption as a mechanism for how they affect care quality [65].

^b^CERQual: Confidence in the Evidence from Reviews of Qualitative Research.

^c^MCQ: multiple-choice questionnaire.

#### Objective 1: Impacts of OCs on Primary Care Quality

##### Safety

In 27% (17/63) of the studies, staff and patients expressed general concerns about the impact of OCs on patient safety, particularly regarding the potential loss of information from patients versus in-person or telephone consultations and how it could lead to misdiagnosis [55]. However, quantitative evidence from 17% (11/63) of the studies did not support these concerns in terms of emergency department attendance rates [92], hospitalizations [70], deaths [88], and other measures [59]. Furthermore, clinicians and patients in 22% (14/63) of the studies believed that OCs improved patient safety, for example, by producing a detailed shared written record of consultations [93] and helping reduce the spread of communicable diseases such as COVID-19 [63].

##### Effectiveness

In 6% (4/63) of the studies, antibiotics were prescribed to patients at a lower rate via OCs compared with in-person consultations [60].

##### Timeliness

In 46% (29/63) of the studies, OCs were perceived as increasing access to primary care services. It was easier and more convenient to make initial contact as patients could submit an OC query at any time without waiting on the phone or attending in person [14]. Once a query was submitted, patients also communicated with health professionals sooner as OCs tended to circumvent the traditional appointment-booking process [57].

##### Efficiency

In total, 52% (33/63) of the studies suggested that the workload decreased for both staff and patients when using OCs. Patient queries were written rather than spoken, incoming phone calls to receptionists were reduced [16], and patient histories did not need manual documentation [93]. Written queries were usually more detailed than when communicated verbally and were received by health care staff asynchronously, thus providing opportunities for more objective examination and more effective triage. Consequently, patient queries could more often be directed to other services or dealt with by other staff members rather than always by physicians [3]. Combined with their remote nature, OCs also gave staff more autonomy over how their work was organized, thus providing efficiency gains such as working from home and control over how to contact a patient rather than defaulting to an in-person consultation [13]. When telephone or in-person consultations were necessary, they were more focused and, therefore, quicker as the staff member could read the patient query before contact [17]. OCs reduced the workload for patients by avoiding the need to telephone their primary care provider to make an appointment, which often entailed long queues [18], and avoiding in-person consultations when possible, which typically involved travel, waiting rooms, and organizing time off work and childcare [15].

In contrast, 46% (29/63) of the studies suggested that OCs increased the workload for staff and patients. Staff described conducting OCs on top of their usual tasks [13] and dealing with them outside normal working hours [19]. They believed that, because OCs increased access to primary care, patients sought help more readily than they would have previously [17], thus creating “supply-induced demand” [108]. Processing OCs also created new administrative work such as filing them to EHRs and deciding whether they required input from a clinician [86]. Workload could also increase for patients if they perceived that entering their query into the OC system was more difficult than explaining it verbally [20].

OCs decreased costs for providers in 32% (20/63) of the studies largely by reducing in-person visits, which have associated expenditures related to staffing and utilities [21]. Patients reported that, owing to their convenience, having access to OCs stopped them from visiting other costly unscheduled care providers [92]. OCs decreased costs for patients in 6% (4/63) of the studies by avoiding in-person visits, which may entail expenses related to travel, unpaid work leave, and childcare [57].

In contrast, OCs increased costs for providers in 14% (9/63) of the studies owing to associated technology costs [63], time required for clinicians to triage patient queries [22], and insufficient reduction of in-person visits or telephone consultations [87].

##### Equitable

In all, 65% (41/63) of the studies suggested that OCs decreased equitable access to care services, as their use varied according to patient characteristics [63]. Conversely, 30% (19/63) of the studies suggested that OCs increased equitable access as they helped particular groups of patients who had previously struggled communicate with their primary care providers [14]. These characteristics are discussed in more detail in the Adopters section.

##### Patient-Centeredness

Although 21% (13/63) of the studies uncovered some patient dissatisfaction with OCs [90], 49% (31/63) found that most patients were at least as satisfied or more satisfied with OCs than with traditional in-person appointments [2]. Patients liked OCs for the aforementioned reasons: they improved access (timeliness), reduced their workload and costs (efficiency), and helped particular groups of patients communicate with their care providers (equitable).

#### Objective 2: How the Impacts of OCs on Primary Care Quality Are Influenced by System Design and Implementation

##### Condition Complexity

In all, 17% (11/63) of the studies suggested that OCs decreased staff workload when used for simple queries that were straightforward to resolve as they were more amenable to completion without needing to contact the patient directly via telephone or in person [5]. Simple queries included those related to administrative tasks, new and recurrent minor acute illnesses, prescriptions, tests, requests for advice, follow-up, and some chronic condition reviews [56]. These queries also decreased health costs as they saved clinicians time, for example, when administrative staff were able to relay messages and there was no direct contact between physician and patient [23]. In all, 11% (7/63) of the studies suggested that OCs increased staff workload and costs when used for complex queries such as those with multiple ill-defined symptoms [17]. These queries generally required verbal dialogue with and physical examination of the patient and were usually converted to telephone or in-person consultations to assess the patient further [23]. Staff felt that this duplicated the number of contacts with the patient for the same query.

##### Technology

In all, 21% (13/63) of the studies showed that, when patients had to use MCQs to input their OC query, it increased both patient and staff workload. Filling out long lists of questions shifted work from the clinician to the patient [20], and staff found them burdensome to read [86]. MCQs limited the amount of detail patients could enter, so staff could not always fully understand their request. This increased workload as they often had to contact the patient to obtain further information [23]. MCQs also asked questions about seemingly “irrelevant” symptoms, which staff were responsible for assessing and following up, diverting attention from the patient’s primary concern [10]. Owing to the restrictive nature of MCQs, patients regularly adapted their responses to obtain the outcome they wanted even when it was not the most appropriate use of resources. For example, reporting their symptoms differently to obtain an in-person consultation when self-care may have been more suitable (“gaming”) [17].

In all, 14% (9/63) of the studies suggested that MCQs could also decrease patient satisfaction. Reasons included the amount of work required to complete them [14], their inflexibility in obtaining the answers patients wanted from their primary care provider [9], and that they could be confusing to navigate [25].

In contrast, 11% (7/63) of the studies suggested that, when patients could primarily report their queries using unstructured free text, it decreased staff workload and increased patient safety. This was because patients were more able to fully describe their query in sufficient detail using their own words, and clinicians did not have to request further information as often [95].

In 10% (6/63) of the studies, two-way written communication within the OC decreased the workload for both staff and patients. The ability to reply to patients in writing meant queries could be answered and follow-up questions could be asked at times convenient to both staff and patients, avoiding lengthy telephone and in-person consultations when appropriate [55]. It was also easier to communicate complex information, for example, by sending educational materials or using preset message templates [95].

In all, 21% (13/63) of the studies highlighted that a lack of integration between the OC system and other core software used by providers increased staff workload. Nonintegration meant that the staff had to go through multiple steps to perform a task, such as when filing an OC to a patient’s EHR [21].

##### Adopters

Patients using OCs were more likely to be female (27/63, 43%) [70], younger (27/63, 43%) [91], and native speakers of the official language of the country they lived in (7/63, 11%) [25] and have a higher socioeconomic status (11/63, 17%) [57] than those not using OCs, thus decreasing equity. In contrast, both staff and patients felt that OCs increased access for particular groups of patients who struggled with traditional consultation methods, thus increasing equity and satisfaction with care. This included patients with mental health conditions who became anxious when speaking to health professionals on the telephone or in person (8/63, 13%) [20]; patients with verbal communication difficulties such as hearing loss who found it easier to communicate in writing (7/63, 11%) [90]; and patients with barriers to attending in-person appointments because of physical disabilities, geography, work commitments, or care responsibilities (8/63, 13%) [23]. In all, 13% (8/63) of the studies suggested that when staff and patients viewed traditional in-person methods as the gold standard, it could lead to resistance in adopting OCs [19].

##### Organization

In all, 8% (5/63) of the studies found that, when OCs were minimally advertised to patients, it understandably led to low rates of adoption [24]. In all, 11% (7/63) of the studies also showed that responding to a patient’s initial OC query quickly led to high patient satisfaction, as it provided an advantage over traditional methods of primary care contact [6]; by definition, this also increased primary care access.

In all, 21% (13/63) of the studies found that the staff workload increased when providers did not integrate OCs into their normal daily workflows. For example, not scheduling time for clinicians to deal with OCs meant that they were done in addition to their normal tasks [93], and not diverting all incoming patient demand via the OC meant that different communication routes were often used for the same issue, thereby duplicating work [5]. In all, 13% (8/63) of the studies suggested that provider workload decreased if sufficient resources were allocated to implementing OCs. This included their initial setup—for example, training to enable staff to more effectively handle OCs [15]—and their ongoing processing—for example, dedicated facilities such as quiet rooms to help staff respond to OCs without distraction [55].

In all, 8% (5/63) of the studies showed that a lack of continuity of care between patients and their known physician negatively affected patient satisfaction. This occurred when any physician could reply to an OC query and patients were not able to specify a physician to whom to address their query [64].

##### Wider System

In all, 10% (6/63) of the studies showed that government policies mandating OC use increased their adoption. Example policies aimed to increase digital modes of contact with primary care in general [87] and minimize in-person contact during the COVID-19 pandemic [63]. In all, 8% (5/63) of the studies demonstrated that a lack of long-term external financial support for OCs limited their sustainability as health care organizations could often not afford to pay their ongoing costs [23].

## Discussion

### Summary of Evidence

This review focused on how OCs affect primary care quality, as defined by Institute of Medicine domains, for patients, providers, and the wider system, as well as which factors, as specified through the NASSS framework, influence this quality. We synthesized qualitative and quantitative evidence from 63 studies conducted in 9 countries covering 31 OC systems described in detail, with wide-ranging functionalities including AI. In all, 41% (26/63) of the studies were published in 2020 onward, and 17% (11/63) were published after the COVID-19 pandemic. Our main findings were that OCs are safe and have positive impacts on care quality, including increased access to care and decreased patient costs. However, they can have conflicting impacts on provider costs, staff and patient workloads, patient satisfaction, and care equity. We found that the impacts OCs have on care quality are determined by the complexity of the patient queries they are used for, the design of the OC technology itself, the characteristics of staff and patient users, the way OCs are implemented by health care providers, and wider health policies.

### Comparison of Findings With Other Reviews

Consistent with previous reviews relevant to OCs, we found a limited demographic of patients using OCs, leading to potential inequitable care [45,46]. We also found that the studies often did not sufficiently explore patients’ perspectives of OCs in depth [46]; only 14% (9/63) of the studies used interview-based methods with an average sample size of 24.5 (SD 10.14). This hampered efforts to understand how such inequities arose.

Contrary to previous reviews, we found that OC impacts on care quality are more complex and nuanced than previously reported [45-47]. For example, we identified mixed findings regarding their impact on workload, patient satisfaction, and equitable care. This contrasts with previous reviews, where OCs only increased [47] or had no impact [45] on workload, decreased patient safety [45,47], and increased inequity [45-47].

These new findings for OCs may be partly explained because 76% (48/63) of the included studies had not been covered by these previous reviews. Although there was some overlap of papers (7/57, 12% of papers [45]; 7/13, 54% of papers [46]; and 4/17, 24% of papers [47]), most did not meet our inclusion criteria as they were either nonempirical (4/57, 7% [45]; 4/13, 31% [46]; and 4/17, 24% [47]), published before 2010 (26/57, 46% [45] and 2/17, 12% [47]), not based on real-world primary care (16/57, 28% [45]; 1/13, 8% [46]; and 6/17, 35% [47]), or did not meet our functional definition of an OC (39/57, 68% [45]; 2/13, 15% [46]; and 6/17, 35% [47]; eg, symptom checkers with no link to a health professional [28]).

By focusing on design and implementation, we identified new ways in which OCs affect primary care quality. For example, we found that, by increasing access, OCs can increase staff workload by creating “supply-induced demand” [17,108] and that they can decrease workload by enabling more focused consultations [17]. Furthermore, as previous reviews often did not analyze the design or implementation of OCs [45-47], we identified influential factors that have not been previously described. For example, although some reviews identified increased workload when clinicians received insufficient patient information via an OC system [46], we found that this was particularly associated with MCQ-based OCs [23]. We identified that allowing patients to describe their queries using unstructured free text had the opposite effect [95] while also having a positive impact on patient safety [55]. Using unstructured free text means that patients can more fully describe their query in addition to allowing them to freely express their ideas, concerns, and expectations, as is common in patient-centered primary care consultations [109].

### Strengths and Limitations

As evidenced by the range of examples in Multimedia Appendix 1, we adopted a fundamental functional definition of OCs rather than relying on the names given to them by the authors of the included studies. When combined with our comprehensive searches across multiple databases and inclusion of gray literature, we identified more empirical studies relevant to OCs than any previous evidence synthesis on the topic [45-47]. Combined with our focus on causal mechanisms, this helped us develop a new and theoretically informed understanding of OCs that has not been previously reported.

As in all systematic reviews, our synthesis is reliant on what the study authors reported. OC features were not always described in sufficient detail to understand how they affected care quality [62]. There was also a lack of patient perspective in the studies, particularly from OC nonusers [4]. We made our literature search strategy as inclusive as possible regarding the different terms used for OCs (Multimedia Appendix 1) but, owing to their wide-ranging nature, it is possible that some papers were missed. We updated our searches between November 2021 and February 2022 to capture more recently published studies but, owing to time constraints, only 1 author (SD) screened these newer papers. This enabled us to capture studies conducted in the context of COVID-19 (11/63, 17% of all included studies).

### Implications for Practice and Research

#### Overview

Our findings show that the impacts of OCs on care quality are complex and can be influenced by the subtle ways in which OCs are designed and implemented. To maximize their benefit for patients and staff, we therefore provide recommendations for OC developers on how systems could be designed, health care organizations on how they can be implemented and used, and researchers on questions and areas for further investigation. They are discussed in the following sections under the high-level themes from objective 2 and summarized in Table 4.

**Table 4:** Implications for online consultation (OC) research and practice.

Theme	Implications		
	OC designers	Health care providers	Researchers
Condition complexity	Help health care providers identify when patients have submitted a query that could be unsuitable for resolution via an OC; for example, a complex condition	Currently, all complex queries should be routed through traditional consultation methods	Can OCs be used for complex queries and, if so, how can they be best adapted to support their resolution? What impact do OCs have on clinical outcomes?
Technology	Primarily allow patients to describe their queries using unstructured free text rather than MCQs^a^ Allow two-way written messages to be sent between staff and patients Guide and support patients to provide sufficient detail about their query Integrate with existing core clinical software systems used by health care organizations Support patients to self-care or signpost them to other services when appropriate Match capacity to demand by limiting the volume of OC queries a primary care provider can receive Support workflow (eg, determining whether OCs need clinical vs administrative input) Assist in triaging patient queries Highlight when patients may require an in-person appointment Explore the potential of using AI^b^ to automate the aforementioned functions	Guide and support patients to provide sufficient detail about their query	Is the additional demand via OCs supply-induced or a previously unmet (and now unmasked) need? How can AI be effectively used in OCs? Fully describe the OC systems studied in detail (eg, using the TIDieR^c^ checklist [110])
Adopters	Involve patients from a variety of backgrounds in designing OC systems to facilitate their adoption	Involve patients from a variety of backgrounds in planning how OCs are implemented Explain and promote the benefits of OCs to staff and patients during their implementation—including increased access for certain patient groups (eg, those with mental health conditions, verbal communication difficulties, and barriers to attending in-person appointments)	What is the experience of patient users and low or nonusers of OCs from a range of backgrounds? Why are patients with different characteristics more or less likely to use OCs? How can patients from different backgrounds be supported to use OCs effectively? Are there other specific patient groups likely to benefit from OCs and why? In what circumstances are in-person consultation methods viewed as the gold standard and why? How are OCs being used after the COVID-19 pandemic?
Organization	Facilitate planning and booking OCs into clinicians’ daily schedules	Widely promote OCs to patients through various channels (eg, mail-out campaigns) Provide sufficient staff training on OCs Divert as much incoming patient demand as possible through OCs Plan OCs into clinicians’ daily schedules Initially respond to patients through written message or phone call as soon as possible on the same day to acknowledge their query	How can OCs most effectively be incorporated into daily workflows? Are OCs suitable for middle-income countries?
Wider system	N/A^d^	Use system-wide policies to increase OC uptake Centralized funding is required to ensure sustainability	What is the long-term experience of policies mandating OC use, particularly in light of the COVID-19 pandemic?

^a^MCQ: multiple-choice questionnaire.

^b^AI: artificial intelligence.

^c^TIDieR: Template for Intervention Description and Replication.

^d^N/A: not applicable.

#### Condition Complexity

It is unclear whether OCs are unsuitable for complex patient queries or whether workflows and procedures can be better organized and OC systems can be better designed to deal with them. Therefore, we recommend that (1) complex conditions are routed through traditional consultation methods (eg, in person and telephone) and (2) further research is conducted on how these types of conditions could be better handled via OCs to ensure that they benefit all patients.

#### Technology

On the basis of existing evidence, we recommend that OC developers (1) allow patients to fully describe their queries using unstructured free text rather than MCQs, (2) support patients in providing sufficient detail in their queries for their primary care provider to respond quickly and safely, (3) allow for two-way written communication between staff and patients, and (4) integrate their solutions with existing core clinical software systems.

Technology design also plays a role in mitigating some of the undesirable outcomes we identified from using OCs, including increasing workload and costs. Increased workload is particularly important as it can lead to a mismatch between patient demand and health care resources, which can in turn threaten patient safety if providers are unable to deal with OCs in an appropriate time frame. A way this could happen is through increased demand—if there are too many OCs submitted by patients and not enough staff to deal with them [55]. Whether this additional demand is a supply-induced [108] or previously unmet (and now unmasked) need was unclear from the studies we included [15] and requires further research. Nevertheless, OC systems could help by (1) supporting patients to self-care or signposting them to other services when appropriate; (2) matching capacity to demand by limiting the number of OC queries that primary care providers can receive from patients; (3) supporting workflow, for example, by determining whether OCs require clinical input to relieve the workload of administrators [86]; (4) assisting in triaging patient queries to reduce the associated costs of solely relying on clinicians for triage [22]; and (5) highlighting when patients may require an in-person appointment to facilitate direct booking to avoid work duplication [23], which may relate to patient query complexity.

**Figure 2:**
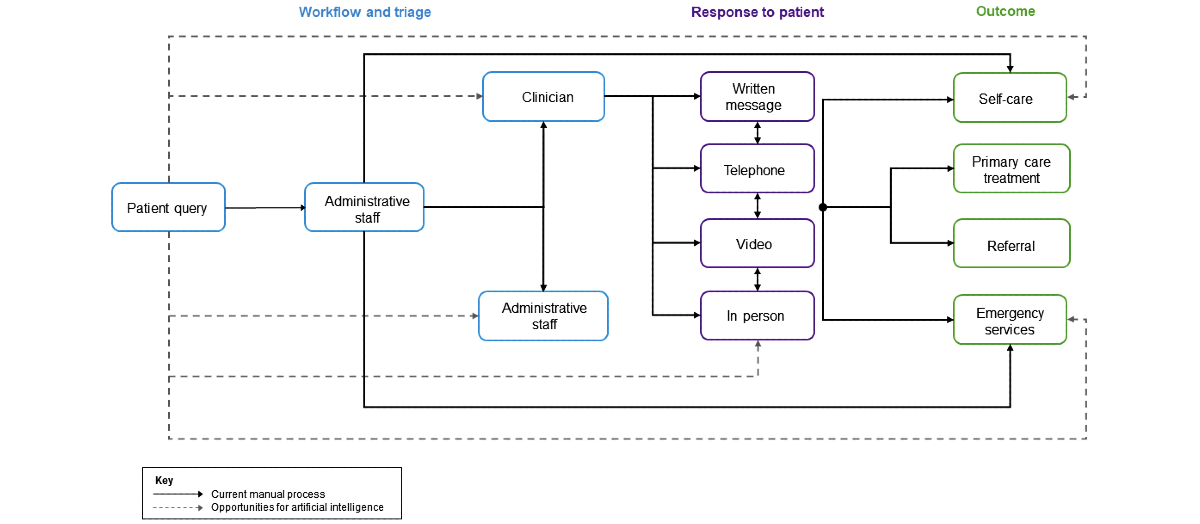
Artificial intelligence opportunities.

According to our definition [82], many of these functions may require AI to be most effective, which should be explored by OC designers (Figure 2). In all, 54% (13/24) of MCQ-based OC systems in our review used AI (Table 1) [54], although largely for other functions rather than the aforementioned ones. Furthermore, AI was usually not the focus of the studies, and we consequently found only low-confidence evidence regarding its use in OCs (Multimedia Appendix 8). Therefore, how AI could be used by OC systems in clinical practice requires further research.

The included papers did not always adequately describe the OC systems studied, limiting our ability to determine how their specific features affected care quality. Future research should describe OC systems in detail so that evaluation findings can be usefully compared, for example, by using the Template for Intervention Description and Replication checklist [110].

#### Adopters

We found inadequate exploration of participant (especially patient) experiences to confidently explain how and why the impacts on care equity arose during OC use. Study authors and health care staff often speculated reasons [18], but this was insufficient to formulate evidence-based hypotheses. Future research should explore the perspectives of patients using (and not using) OCs from a wide range of backgrounds using in-depth qualitative techniques such as interview-based methods. Patients from a variety of backgrounds should be involved in how OC systems are designed and help plan how they are implemented in practice.

Staff and patients resisted adopting OCs when they viewed traditional in-person consultation methods as the gold standard. Although this was understandable for complex queries [17], it was unclear whether other factors also influenced this view. Future research should address this evidence gap, particularly as COVID-19 has made remote consultations more commonplace [49]. In the meantime, this perception could be challenged by explaining the benefits of OCs found in our review to prospective users [111].

#### Organization

For patients and staff to experience the benefits of OCs, they must be widely promoted to patients as a route for them to contact their primary care provider. This can happen through various channels, such as mail-out campaigns (eg, via SMS text message) or by verbally mentioning OCs when in contact with patients (eg, when receptionists speak to patients on the telephone).

To minimize workload associated with OCs, we recommend that organizations (1) allocate sufficient resources to both setting up and processing them, including the provision of training on how to use OCs, and to staff and facilities (eg, computers and rooms) to deal with them; (2) divert as much incoming patient demand as possible through the system to avoid duplication and increase the proportion of patient contacts that benefit from OCs; and (3) incorporate OCs into daily work patterns by scheduling protected time for staff to deal with them to ensure that they do not become additional tasks to complete on top of their normal work.

Our findings show that providers can increase access and patient satisfaction by responding quickly to OCs, although the definitions of what this involved were unclear. We recommend providing an initial response to patients’ OC queries as soon as possible on the same day—either through written message or telephone call. This does not mean that the entire query needs to be resolved at this point, only that initial contact has been made and the query has been acknowledged.

We included studies from 9 countries, all of which were high-income Western countries. Owing to their remote nature, OCs may play a role in middle-income countries where there are isolated communities and fewer health care staff per head of population. However, further research is required to understand how their technological and financial barriers could be overcome.

#### Wider System

Governmental policies to promote OCs are effective in increasing adoption, although centralized funding is needed to sustain their use. It is unclear what the long-term experience of such policies is from the papers we included, particularly in response to those relating to the COVID-19 pandemic.

### Conclusions

This is the first theoretically informed synthesis of empirical research on OCs in primary care and uniquely includes studies conducted during the COVID-19 pandemic. It contributes new knowledge that OCs are safe and have positive impacts on care quality, including increased access to primary care and decreased patient costs. However, they are also complex and often produce conflicting impacts on provider costs, staff and patient workloads, patient satisfaction, and care equity. Some of these are unintended and conflict with the promotion of OCs by policy makers as a way to address already increasing workload and decreasing workforce capacity in primary care [31-36]. Unlike previous evidence syntheses on the topic, we have shown that negative impacts on care quality of OCs can be mitigated through appropriate system design (eg, free text formats and two-way written communication), incorporation of advanced technologies (eg, AI), and integration into technical infrastructure (eg, EHRs) and organizational workflows (eg, timely responses). Since the advent of COVID-19, OCs have become indispensable, although further engineering and implementation research is required to realize their full benefits.

## Appendix

Multimedia Appendix 1 Terms used by the included studies for online consultations.

Multimedia Appendix 2 Search terms.

Multimedia Appendix 3 Data extraction form.

Multimedia Appendix 4 Descriptive summary of the included studies.

Multimedia Appendix 5 Description of the online consultation systems studied.

Multimedia Appendix 6 Quality appraisal of the included studies using the Mixed Methods Appraisal Tool.

Multimedia Appendix 7 Low-confidence findings for objective 1.

Multimedia Appendix 8 Low-confidence findings for objective 2.

Multimedia Appendix 9 Outcomes of online consultations in primary care (with exemplar data).

Multimedia Appendix 10 How outcomes of online consultations in primary care are influenced by system design and implementation (with exemplar data).

## Abbreviations

AI: artificial intelligence
EHR: electronic health record
MCQ: multiple-choice questionnaire
MMAT: Mixed Methods Appraisal Tool
NASSS: nonadoption, abandonment, scale-up, spread, and sustainability framework
OC: online consultation
PRISMA: Preferred Reporting Items for Systematic Reviews and Meta-Analyses
