# Supplementary material for: Understanding How the Design and Implementation of Online Consultations Affect Primary Care Quality: Systematic Review of Evidence With Recommendations for Designers, Providers, and Researchers
Source: J Med Internet Res. 2022 Oct 24;24(10):e37436. doi: 10.2196/37436 (PMC9621309; doi:10.2196/37436)
Supplement: Multimedia Appendix 1 [file jmir_v24i10e37436_app1.docx]

**Appendix 1: Terms used by included studies (n=63) for online consultations**

| **Term** | **Number of publications using each term (%)** | **References** |
| --- | --- | --- |
| Triage system based on a symptom-checker | 1 (2) | [76] |
| Asynchronous chat | 1 (2) | [45] |
| Digital care | 1 (2) | [104] |
| Digital health consultations | 1 (2) | [42] |
| Digital healthcare platform | 1 (2) | [65] |
| Digital primary (health) care | 3 (5) | [27,90,91] |
| Digital doctor reception | 1 (2) | [28] |
| Digital communication system | 1 (2) | [38] |
| Digital chat-based communication system | 1 (2) | [44] |
| Electronic consultations/e-consultation/e-consult | 16 (25) | [37,39,43,46,64,71,81-84,86-89,102,105] |
| eVisit/e-Visit | 20 (31) | [30,34,35,36,70,72,73,75,79,80,92-94,97,98,100,101,103,138,145] |
| Online consultation | 4 (6) | [47,63,69,69,95] |
| Online visit | 1 (2) | [99] |
| Online triage | 1 (2) | [74] |
| Self-triage/self-scheduling | 1 (2) | [29] |
| Teleconsultation/Telehealth | 3 (5) | [26,31,33] |
| Virtual consulting | 1 (2) | [41] |
| Virtual visit | 5 (8) | [25,32,40,78,96] |

**References**

1. Ipsos MORI, York Health Economics Consortium. Evaluation of Babylon GP at Hand: Final evaluation report. London2019 [cited 2020 1 Sept]; Available from: <https://www.hammersmithfulhamccg.nhs.uk/media/156123/Evaluation-of-Babylon-GP-at-Hand-Final-Report.pdf>.

2. Cajander Å, Larusdottir M, Hedström G. The effects of automation of a patient-centric service in primary care on the work engagement and exhaustion of nurses. Qual User Exp. 2020 19 Sept;5(1):9. doi: 10.1007/s41233-020-00038-x.

3. Ekman B, Thulesius H, Wilkens J, Lindgren A, Cronberg O, Arvidsson E. Utilization of digital primary care in Sweden: Descriptive analysis of claims data on demographics, socioeconomics, and diagnoses. Int J Med Inform. 2019;127:134-40. doi: 10.1016/j.ijmedinf.2019.04.016.

4. Landgren S, Cajander Å. Non-use of Digital Health Consultations Among Swedish Elderly Living in the Countryside. Front Public Health. 2021 2021-September-10;9(1323). doi: 10.3389/fpubh.2021.588583.

5. Peber E, Wästfelt E. Impact of digi-physical healthcare. [Master Thesis] Sweden: Lund University; 2020 [cited 2020 1 Sept]; Available from: <https://lup.lub.lu.se/student-papers/search/publication/9015211>.

6. Johansson A, Larsson M, Ivarsson B. Patients' Experiences With a Digital Primary Health Care Concept Using Written Dialogues: A Pilot Study. J Prim Care Community Health. 2020 01 Jan;11:2150132720910564. doi: 10.1177/2150132720910564.

7. Health Innovation Manchester. GM Digital First Primary Care: Patient and public insights: Workshop results. Report. Manchester: Health Innovation Manchester, 2021 Sept 2021. Report No.: 1 Contract No.: 1 Sept.

8. Health Innovation Manchester. Greater Manchester digital primary care insight full report: Key findings from engagement June-July 2020. [Report] 2020 [cited 2021 1 Oct]; Available from: <https://healthinnovationmanchester.com/wp-content/uploads/2020/12/PCIE-DPC-Full-report-FINAL-20.10.20-1.pdf>.

9. Johansson A, Larsson M, Ivarsson B. General Practitioners' Experiences of Digital Written Patient Dialogues: A Pilot Study Using a Mixed Method. J Prim Care Community Health. 2020 Mar;11. PMID: WOS:000523514400001. doi: 10.1177/2150132720909656.

10. Eldh AC, Sverker A, Bendtsen P, Nilsson E. Health Care Professionals' Experience of a Digital Tool for Patient Exchange, Anamnesis, and Triage in Primary Care: Qualitative Study. JMIR Hum Factors. 2020;7(4):e21698. PMID: 33315014. doi: 10.2196/21698.

11. Nilsson E, Sverker A, Bendtsen P, Eldh AC. A Human, Organization, and Technology Perspective on Patients’ Experiences of a Chat-Based and Automated Medical History–Taking Service in Primary Health Care: Interview Study Among Primary Care Patients. J Med Internet Res. 2021 2021/10/18;23(10):e29868. doi: 10.2196/29868.

12. Atherton H, Brant H, Ziebland S, Bikker A, Campbell J, Gibson A, et al. The potential of alternatives to face-to-face consultation in general practice, and the impact on different patient groups: a mixed-methods case study. Health Serv Deliv Res 2018;6(20). PMID: 29889485. doi: 10.3310/hsdr06200.

13. Bertelsen P, Petersen L. Danish Citizens and General Practitioners' Use of ICT for their Mutual Communication. In: Sarkar I.N, Georgiou A, Mazzoncini de Azevedo Marques P, editors. MEDINFO 2015: eHealth-enabled Health; August; São Paulo, Brazil: IOS Press; 2015. p. 376-9.

14. Carter M, Fletcher E, Sansom A, Warren FC, Campbell JL. Feasibility, acceptability and effectiveness of an online alternative to face-to-face consultation in general practice: a mixed-methods study of webGP in six Devon practices. BMJ Open. 2018 May;8(2). PMID: WOS:000433129800107. doi: 10.1136/bmjopen-2017-018688.

15. Cowie J, Calveley E, Bowers G, Bowers J. Evaluation of a digital consultation and self-care advice tool in primary care: A multi-methods study. Int J Environ Res Public Health. 2018 02 May;15 (5) (896). doi: 10.3390/ijerph15050896.

16. NHS England. Prime Minister’s Challenge Fund: Improving Access to General Practice First Evaluation Report. NHS England; 2015 [updated October; cited 2020 1 Sept]; Available from: <https://www.england.nhs.uk/wp-content/uploads/2015/10/pmcf-wv-one-eval-report.pdf>.

17. Fagerlund AJ, Holm IM, Zanaboni P. General practitioners' perceptions towards the use of digital health services for citizens in primary care: A qualitative interview study. BMJ Open. 2019 01 May;9 (5)(e028251). doi: 10.1136/bmjopen-2018-028251.

18. Farr M, Banks J, Edwards HB, Northstone K, Bernard E, Salisbury C, et al. Implementing online consultations in primary care: a mixed-method evaluation extending normalisation process theory through service co-production. BMJ Open. 2018 Mar;8(3). PMID: WOS:000433881200172. doi: 10.1136/bmjopen-2017-019966.

19. Nijland N, van Gemert-Pijnen J, Kelders SM, Brandenburg BJ, Seydel ER. Evaluation of the use of an "ask-the-expert" e-consultation service for support on health-related requests. Second International Conference on eHealth, Telemedicine, and Social Medicine; 10-16 Feb. 2010: IEEE; 2010. p. 72-6.

20. Zanaboni P, Fagerlund AJ. Patients' use and experiences with e-consultation and other digital health services with their general practitioner in Norway: Results from an online survey. BMJ Open. 2020 17 Jun;10 (6) (e034773). doi: 10.1136/bmjopen-2019-034773.

21. Matheson C. Implementation of WebGP and Econsultations in Wessex GP Practices: Interim Update Report. Southampton, UK: Centre of Implementation Science; 2016 [updated June; cited 2020 1 Sept]; Available from: <https://eprints.soton.ac.uk/397189/1/__soton.ac.uk_ude_personalfiles_users_cbm1a13_mydocuments_CBM%2520WebGP%2520update%252021062016IR.pdf>.

22. Leung K, Qureshi S. Managing high frequency users of an electronic consultation system in primary care: a quality improvement project. BMJ Open Qual. 2021;10(2). PMID: WOS:000663456800001. doi: 10.1136/bmjoq-2020-001310.

23. Nijhof D, Ingram A, Ochieng R, Roberts E-J, Poulton B, Ochieng B. Examining GP online consultation in a primary care setting in East Midlands, UK. BMC Health Serv Res. 2021 2021/09/30;21(1):1030. doi: 10.1186/s12913-021-07039-2.

24. Atherton H, Brant H, Ziebland S, Bikker A, Campbell J, Gibson A, et al. Alternatives to the face-to-face consultation in general practice: Focused ethnographic case study. Br J Gen Pract. 2018 April;68(669):e293-e300. doi: 10.3399/bjgp18X694853.

25. Banks J, Farr M, Salisbury C, Bernard E, Northstone K, Edwards H, et al. Use of an electronic consultation system primary care: a qualitative interview study. Br J Gen Pract. 2018 Jan;68(666):E1-E8. PMID: WOS:000425962100001. doi: 10.3399/bjgp17X693509.

26. Edwards HB, Marques E, Hollingworth W, Horwood J, Farr M, Bernard E, et al. Use of a primary care online consultation system, by whom, when and why: Evaluation of a pilot observational study in 36 general practices in South West England. BMJ Open. 2017;7(11). doi: 10.1136/bmjopen-2017-016901.

27. Murphy M, Scott LJ, Salisbury C, Turner A, Scott A, Denholm R, et al. Implementation of remote consulting in UK primary care following the COVID-19 pandemic: a mixed-methods longitudinal study. Br J Gen Pract. 2021;71(704):e166-e77. doi: 10.3399/bjgp.2020.0948.

28. Bishop TF, Press MJ, Mendelsohn JL, Casalino LP. Electronic communication improves access, but barriers to its widespread adoption remain. Health Aff. 2013;32(8):1361-7. doi: 10.1377/hlthaff.2012.1151.

29. Hertzog R, Johnson J, Smith J, McStay FW, da Graca B, Haneke T, et al. Diagnostic Accuracy in Primary Care E-Visits: Evaluation of a Large Integrated Health Care Delivery System's Experience. Mayo Clin Proc. 2019;94(6):976-84. doi: 10.1016/j.mayocp.2019.02.011.

30. Jung CM, Padman R. Virtualized healthcare delivery: Understanding users and their usage patterns of online medical consultations. Int J Med Inform. 2014 Dec;83(12):901-14. PMID: WOS:000346052600004. doi: 10.1016/j.ijmedinf.2014.08.004.

31. Padman R, Shevchik G, Paone S, Dolezal C, Cervenak J. eVisit: A Pilot Study of a New Kind of Healthcare Delivery. In: Safran C, Reti S, Marin HF, editors. Medinfo 2010, Pts I and Ii2010. p. 262-6.

32. Jung C, Padman R, Shevchik G, Paone S. Who are portal users vs. early e-Visit adopters? A preliminary analysis. AMIA Annu Symp Proc. 2011:1070-9. PMID: 22195168.

33. Murray MA, Penza KS, Myers JF, Furst JW, Pecina JL. Comparison of eVisit Management of Urinary Symptoms and Urinary Tract Infections with Standard Care. Telemed J E Health. 2020 2020/05/01;26(5):639-44. doi: 10.1089/tmj.2019.0044.

34. North F, Crane SJ, Chaudhry R, Ebbert JO, Ytterberg K, Tulledge-Scheitel SM, et al. Impact of Patient Portal Secure Messages and Electronic Visits on Adult Primary Care Office Visits. Telemed J E Health. 2014 2014/03/01;20(3):192-8. doi: 10.1089/tmj.2013.0097.

35. North F, Crane SJ, Stroebel RJ, Cha SS, Edell ES, Tulledge-Scheitel SM. Patient-generated secure messages and eVisits on a patient portal: are patients at risk? J Am Med Inform Assoc. 2013;20(6):1143-9. doi: 10.1136/amiajnl-2012-001208.

36. Peabody MR, Dai M, Turner K, Peterson LE, Mainous AG. Prevalence and Factors Associated with Family Physicians Providing E-Visits. J Am Board Fam Med. 2019;32(6):868. doi: 10.3122/jabfm.2019.06.190081.

37. Penza KS, Murray MA, Myers JF, Furst JW, Pecina JL. Management of Acute Sinusitis via e-Visit. Telemed J E Health. 2021. doi: 10.1089/tmj.2020.0047.

38. Penza KS, Murray MA, Pecina JL, Myers JF, Furst JW. Electronic Visits for Minor Acute Illnesses: Analysis of Patient Demographics, Prescription Rates, and Follow-Up Care Within an Asynchronous Text-Based Online Visit. Telemed J E Health. 2018 2018/03/01;24(3):210-5. doi: 10.1089/tmj.2017.0091.

39. Entezarjou A, Bolmsjö BB, Calling S, Midlöv P, Milos Nymberg V. Experiences of digital communication with automated patient interviews and asynchronous chat in Swedish primary care: a qualitative study. BMJ Open. 2020;10(7):e036585. doi: 10.1136/bmjopen-2019-036585.

40. Entezarjou A, Bonamy A-KE, Benjaminsson S, Herman P, Midlöv P. Human- Versus Machine Learning–Based Triage Using Digitalized Patient Histories in Primary Care: Comparative Study. JMIR Med Inform. 2020;8(9):e18930. PMID: 32880578. doi: 10.2196/18930.

41. Mehrotra A, Paone S, Martich GD, Albert SM, Shevchik GJ. A comparison of care at e-visits and physician office visits for sinusitis and urinary tract infection. JAMA Intern Med. 2013;173(1):72-4. PMID: 23403816. doi: 10.1001/2013.jamainternmed.305.

42. Player M, O’Bryan E, Sederstrom E, Pinckney J, Diaz V. Electronic Visits For Common Acute Conditions: Evaluation Of A Recently Established Program. Health Aff. 2018 2018/12/01;37(12):2024-30. doi: 10.1377/hlthaff.2018.05122.

43. Bavafa H, Hitt LM, Terwiesch C. The Impact of E-Visits on Visit Frequencies and Patient Health: Evidence from Primary Care. Manage Sci. 2018;64(12):5461-80. PMID: 33033417. doi: 10.1287/mnsc.2017.2900.

44. Adamson SC, Bachman JW. Pilot Study of Providing Online Care in a Primary Care Setting. Mayo Clin Proc. 2010;85(8):704-10. doi: 10.4065/mcp.2010.0145.

45. Andersen KN, Nielsen JA, Kim S. Use, cost, and digital divide in online public health care: lessons from Denmark. Transforming Government: People, Process and Policy. 2019;13(2):197-211. doi: 10.1108/TG-06-2018-0041.

46. Mehrotra A, Paone S, Martich GD, Albert SM, Shevchik GJ. Characteristics of Patients Who Seek Care via eVisits Instead of Office Visits. Telemed J E Health. 2013 2013/07/01;19(7):515-9. doi: 10.1089/tmj.2012.0221.

47. Albert SM, Shevchik GJ, Paone S, Martich GD. Internet-based medical visit and diagnosis for common medical problems: experience of first user cohort. Telemed J E Health. 2011 May;17(4):304-8. PMID: 21457013. doi: 10.1089/tmj.2010.0156.

48. Casey M, Shaw S, Swinglehurst D. Experiences with online consultation systems in primary care: Case study of one early adopter site. Br J Gen Pract. 2017 November;67(664):e736-e43. doi: 10.3399/bjgp17X693137.

49. NHS England, NHS Improvement. Online consultations research: Summary research findings. NHS England and NHS Improvement; 2019 [updated February; cited 2020 1 Sept]; Available from: <https://www.england.nhs.uk/wp-content/uploads/2019/09/online-consultations-reserach-summary-of-findings.pdf>.

50. Lawless M, Wright E, Davidson J. A collaborative approach to improving patient access in general practice: Impact of three different pilot schemes in 12 general practices in Greenwich. London J Prim Care. 2016;8(4):56-65. doi: 10.1080/17571472.2016.1173946.

51. Turner A, Morris R, Rakhra D, Stevenson F, McDonagh L, Hamilton F, et al. Unintended consequences of online consultations: a qualitative study in UK primary care. Br J Gen Pract. 2022;72(715):e128. doi: 10.3399/BJGP.2021.0426.

52. Rohrer JE, Angstman KB, Adamson SC, Bernard ME, Bachman JW, Morgan ME. Impact of online primary care visits on standard costs: a pilot study. Popul Health Manag. 2010 Apr;13(2):59-63. PMID: 20415617. doi: 10.1089/pop.2009.0018.

53. Eccles A, Hopper M, Turk A, Atherton H. Patient use of an online triage platform: a mixed-methods retrospective exploration in UK primary care. Br J Gen Pract. 2019 May;69(682):E336-E44. PMID: WOS:000510826900006. doi: 10.3399/bjgp19X702197.

54. Judson TJ, Odisho AY, Neinstein AB, Chao J, Williams A, Miller C, et al. Rapid design and implementation of an integrated patient self-triage and self-scheduling tool for COVID-19. J Am Med Inform Assoc. 2020;27(6):860-6. PMID: 32267928. doi: 10.1093/jamia/ocaa051.

55. Fernández OS, Seguí FL, Vidal-Alaball J, Bonet Simo JM, Vian OH, Cabo PR, et al. Primary Care Doctor Characteristics That Determine the Use of Teleconsultations in the Catalan Public Health System: Retrospective Descriptive Cross-Sectional Study. JMIR Med Inform. 2020 2020/2/1;8(1):e16484. doi: 10.2196/16484.

56. López Seguí F, Walsh S, Solans O, Adroher Mas C, Ferraro G, García-Altés A, et al. Teleconsultation Between Patients and Healthcare Professionals in the Catalan Primary Care Service: Message Annotation Analysis in a Retrospective Cross-Sectional Study. J Med Internet Res. 2020 Sep 17;22(9)(e19149). doi: 10.2196/19149.

57. López Seguí F, Vidal-Alaball J, Sagarra Castro M, García-Altés A, García Cuyàs F. General Practitioners’ Perceptions of Whether Teleconsultations Reduce the Number of Face-to-face Visits in the Catalan Public Primary Care System: Retrospective Cross-Sectional Study. J Med Internet Res. 2020 2020/3/16;22(3):e14478. doi: 10.2196/14478.

58. Wilson G, Currie O, Bidwell S, Saeed B, Dowell A, Halim AA, et al. Empty waiting rooms: the New Zealand general practice experience with telehealth during the COVID-19 pandemic. N Z Med J. 2021 09 Jul;134(1537):89-101. PMID: 34239148.

59. Kelley LT, Phung M, Stamenova V, Fujioka J, Agarwal P, Onabajo N, et al. Exploring how virtual primary care visits affect patient burden of treatment. Int J Med Inform. 2020 2020/09/01/;141:104228. doi: <https://doi.org/10.1016/j.ijmedinf.2020.104228>.

60. McGrail KM, Ahuja MA, Leaver CA. Virtual Visits and Patient-Centered Care: Results of a Patient Survey and Observational Study. J Med Internet Res. 2017 2017/05/26;19(5):e177. doi: 10.2196/jmir.7374.

61. Stamenova V, Agarwal P, Kelley L, Fujioka J, Nguyen M, Phung M, et al. Uptake and patient and provider communication modality preferences of virtual visits in primary care: a retrospective cohort study in Canada. BMJ Open. 2020;10(7):e037064. doi: 10.1136/bmjopen-2020-037064.

62. Tarn DM, Hintz C, Mendez-Hernandez E, Sawlani SP, Bholat MA. Using virtual visits to care for primary care patients with COVID-19 symptoms. J Am Board Fam Med. 2021 February;34:S147-S51. doi: <https://doi.org/10.3122/jabfm.2021.S1.200241>.

63. Johnson KM, Dumkow LE, Burns KW, Yee MA, Egwuatu NE. Comparison of Diagnosis and Prescribing Practices Between Virtual Visits and Office Visits for Adults Diagnosed With Sinusitis Within a Primary Care Network. Open forum infectious diseases. 2019;6(9):ofz393-ofz. PMID: 31660415. doi: 10.1093/ofid/ofz393.
