# Supplementary material for: Understanding How the Design and Implementation of Online Consultations Affect Primary Care Quality: Systematic Review of Evidence With Recommendations for Designers, Providers, and Researchers
Source: J Med Internet Res. 2022 Oct 24;24(10):e37436. doi: 10.2196/37436 (PMC9621309; doi:10.2196/37436)
Supplement: Multimedia Appendix 2 [file jmir_v24i10e37436_app2.doc]

**Appendix 2: Search terms**

| **Database searched** | **Date of Search** | **Search Terms*** | **Filters / Limiters applied** |
| --- | --- | --- | --- |
| Ovid Medline | 30/06/2020 | S1: triage.mp. or Triage/; S2: e-triage; S3: Diagnostic Self Evaluation/ or Self-triage.mp; S4: 1 OR 2 OR 3; S5: consultation.mp; S6: Remote Consultation/ or e-consultation.mp.; S7: S5 OR S6; S8: technology.mp. or Technology/ or Information Technology; S9: electronic.mp. or Electronics/; S10: Digital.mp.; S11: Online.mp.; S12: Mobile Applications/; S13: 8 or 9 or 10 or 11 or 12; S14: primary care.mp. or Primary Health Care/; S15: general practice.mp. or General Practice/; S16: family practice.mp. or Family Practice/; S17: 14 OR 15 OR 16; S18: S4 AND S13; S19: S7 AND S13; S20: S18 AND S17; S21: S19 AND S17; S22: S20 OR S21 | 2010 - CURRENT |
| Ovid Embase | 14/07/2020 | S1: triage.mp. or Triage/; S2: e-triage; S3: Diagnostic Self Evaluation/ or Self-triage.mp; S4: 1 OR 2 OR 3; S5: consultation.mp; S6: Remote Consultation/ or e-consultation.mp.; S7: S5 OR S6; S8: technology.mp. or Technology/ or Information Technology; S9: electronic.mp. or Electronics/; S10: Digital.mp.; S11: Online.mp.; S12: Mobile Applications/; S13: 8 or 9 or 10 or 11 or 12; S14: primary care.mp. or Primary Health Care/; S15: general practice.mp. or General Practice/; S16: family practice.mp. or Family Practice/; S17: 14 OR 15 OR 16; S18: S4 AND S13; S19: S7 AND S13; S20: S18 AND S17; S21: S19 AND S17; S22: S20 OR S21 | 2010 - CURRENT |
| Web of Science (Core Collection) | 21/07/2020 | S1: triage; S2: e-triage; S3: self-triage; S4: "Diagnostic Self Evaluation"; S5: S1 OR S2 OR S3 OR S4; S6: Consultation; S7: "Remote Consultation"; S8: E-consult*; S9: S6 OR S7 OR S8; S10: Technolog*; S11: "Information Technolog*"; S12: Electronic*; S13: Digital; S14: Online; S15: Mobile Phone app*; S16: S10 OR S11 OR S12 OR S13 OR S14 OR S15; S17: "Primary care"; S18: "Primary health care"; S19: "General practi*"; S20: "Family practi*"; S21: S17 OR S18 OR S19 OR S20; S22: S5 AND S16; S23: S9 AND S16; S24: S22 AND S21; S25: S23 AND S21; S26: S24 OR S25 | 2010 - CURRENT |
| Scopus | 23/07/2020 | S1: triage; S2: e-triage; S3: self-triage; S4: "Diagnostic Self Evaluation"; S5: S1 OR S2 OR S3 OR S4; S6: Consultation; S7: "Remote Consultation"; S8: E-consult*; S9: S6 OR S7 OR S8; S10: Technolog*; S11: "Information Technolog*"; S12: Electronic*; S13: Digital; S14: Online; S15: Mobile Phone app*; S16: S10 OR S11 OR S12 OR S13 OR S14 OR S15; S17: "Primary care"; S18: "Primary health care"; S19: "General practi*"; S20: "Family practi*"; S21: S17 OR S18 OR S19 OR S20; S22: S5 AND S16; S23: S9 AND S16; S24: S22 AND S21; S25: S23 AND S21; S26: S24 OR S25 | 2010 - CURRENT |

* Search terms were refined during preliminary scoping search experiments to achieve balance between sensitivity and precision in identifying papers that met our inclusion criteria. Terms using the prefix ‘tele’ such as ‘telemedicine’ and ‘telehealth’ were ultimately dropped due to low precision as they often described stand-alone video conferencing or telephony systems. Despite not including ‘tele’ search terms we still screened papers using them, and ultimately included 11: three described online consultations as ‘teleconsultations’ [26,31,33], one described them as ‘telehealth’ [41], and seven were published in journals with ‘telemedicine’ in their title [34,35,64,79,93,100,103].

**References**

1. Fernández OS, Seguí FL, Vidal-Alaball J, Bonet Simo JM, Vian OH, Cabo PR, et al. Primary Care Doctor Characteristics That Determine the Use of Teleconsultations in the Catalan Public Health System: Retrospective Descriptive Cross-Sectional Study. JMIR Med Inform. 2020 2020/2/1;8(1):e16484. doi: 10.2196/16484.

2. López Seguí F, Walsh S, Solans O, Adroher Mas C, Ferraro G, García-Altés A, et al. Teleconsultation Between Patients and Healthcare Professionals in the Catalan Primary Care Service: Message Annotation Analysis in a Retrospective Cross-Sectional Study. J Med Internet Res. 2020 Sep 17;22(9)(e19149). doi: 10.2196/19149.

3. López Seguí F, Vidal-Alaball J, Sagarra Castro M, García-Altés A, García Cuyàs F. General Practitioners’ Perceptions of Whether Teleconsultations Reduce the Number of Face-to-face Visits in the Catalan Public Primary Care System: Retrospective Cross-Sectional Study. J Med Internet Res. 2020 2020/3/16;22(3):e14478. doi: 10.2196/14478.

4. Wilson G, Currie O, Bidwell S, Saeed B, Dowell A, Halim AA, et al. Empty waiting rooms: the New Zealand general practice experience with telehealth during the COVID-19 pandemic. N Z Med J. 2021 09 Jul;134(1537):89-101. PMID: 34239148.

5. Nijland N, van Gemert-Pijnen J, Kelders SM, Brandenburg BJ, Seydel ER. Evaluation of the use of an "ask-the-expert" e-consultation service for support on health-related requests. Second International Conference on eHealth, Telemedicine, and Social Medicine; 10-16 Feb. 2010: IEEE; 2010. p. 72-6.

6. Murray MA, Penza KS, Myers JF, Furst JW, Pecina JL. Comparison of eVisit Management of Urinary Symptoms and Urinary Tract Infections with Standard Care. Telemed J E Health. 2020 2020/05/01;26(5):639-44. doi: 10.1089/tmj.2019.0044.

7. North F, Crane SJ, Chaudhry R, Ebbert JO, Ytterberg K, Tulledge-Scheitel SM, et al. Impact of Patient Portal Secure Messages and Electronic Visits on Adult Primary Care Office Visits. Telemed J E Health. 2014 2014/03/01;20(3):192-8. doi: 10.1089/tmj.2013.0097.

8. Penza KS, Murray MA, Myers JF, Furst JW, Pecina JL. Management of Acute Sinusitis via e-Visit. Telemed J E Health. 2021. doi: 10.1089/tmj.2020.0047.

9. Penza KS, Murray MA, Pecina JL, Myers JF, Furst JW. Electronic Visits for Minor Acute Illnesses: Analysis of Patient Demographics, Prescription Rates, and Follow-Up Care Within an Asynchronous Text-Based Online Visit. Telemed J E Health. 2018 2018/03/01;24(3):210-5. doi: 10.1089/tmj.2017.0091.

10. Mehrotra A, Paone S, Martich GD, Albert SM, Shevchik GJ. Characteristics of Patients Who Seek Care via eVisits Instead of Office Visits. Telemed J E Health. 2013 2013/07/01;19(7):515-9. doi: 10.1089/tmj.2012.0221.

11. Albert SM, Shevchik GJ, Paone S, Martich GD. Internet-based medical visit and diagnosis for common medical problems: experience of first user cohort. Telemed J E Health. 2011 May;17(4):304-8. PMID: 21457013. doi: 10.1089/tmj.2010.0156.
