# Supplementary material for: Understanding How the Design and Implementation of Online Consultations Affect Primary Care Quality: Systematic Review of Evidence With Recommendations for Designers, Providers, and Researchers
Source: J Med Internet Res. 2022 Oct 24;24(10):e37436. doi: 10.2196/37436 (PMC9621309; doi:10.2196/37436)
Supplement: Multimedia Appendix 5 [file jmir_v24i10e37436_app5.doc]

**Appendix 5: Description of Online Consultation systems** studied

| **Software program (if stated)** | **Description** | **Relevant features from Table 1** | **Papers** |
| --- | --- | --- | --- |
| eConsult (previously WebGP) | Online platform which includes symptom checker, pharmacy advice, administrative service, and e-consultation. To submit an e-consultation, patients complete an online form to provide a structured medical account of their condition. Once received, clinicians contact the patient, usually by telephone. | - One-way communication only - Multiple choice questionnaires with optional free-text - No integration - No artificial intelligence | [71,82-84,86-89,125] |
| Tele-Doc | A condition-specific online questionnaire with five sections, all of which must be completed. Received by the practice as an email attachment and dealt with according to locally specified organisational routines. | - One-way communication only - Multiple choice questionnaires with optional free-text - No integration - No artificial intelligence | [69] |
| eVisit or UPMC HealthTrak (MyChart/Epic Corporation) | Patient selects a condition, completes the structured documentation and submits the eVisit via the portal. Support staff review and forward the submission to a physician. | - Two-way communication - Multiple choice questionnaires with optional free-text - Electronic health record integration - Adapting patient questions during query submission - Pre-programmed logic | [75,92,93,101,103] |
| Flow Platform (Doctrin AB) | Patients choose among a prespecified list of queries and access a structured patient interviewing software, where patients can briefly formulate ideas, concerns, and expectations, and subsequently answer a symptom-specific multiple-choice survey. The software selects suitable subsequent survey questions based on the patient’s answers. A healthcare provider (usually a nurse) then contacts the patient. | - Two-way communication - Multiple choice questionnaires with optional free-text - No integration - Adapting patient questions during query submission - Artificial intelligence method: unclear | [30,36,38,65] |
| Mayo Clinic Patient Portal | Computer directed interview resulting in a structured message to the provider with information pertinent to the new symptom. Providers respond to e-visits by text (secure message) or telephone. | - Two-way communication - Multiple choice questionnaires only - No integration - No artificial intelligence | [72,100] |
| Intuit | Patients enter reported problem (e.g. back pain) and then answer questions one at a time. Questions branch such that history is organized into a readable clinical format. Including validated diagnostic instruments, photo uploads, and additional free-text following completion of questionnaire. | - Two-way communication - Multiple choice questionnaires with optional free-text - No integration - Adapting patient questions during query submission - Pre-programmed logic | [94] |
| Patient portal self-triage and self-scheduling tool for COVID-19 | Automated symptom assessment, triage, and appointment scheduling for patients with concerning symptoms or questions about COVID-19. After answering a series of questions, patients are segmented into risk categories and directed to 1 of 4 endpoints. Basic demographic information is automatically populated from the patient’s medical record. Clinicians only review encounters if patients call the triage hotline or clinic or make an appointment. | - One-way communication only - Multiple choice questionnaires only - Electronic health record integration - Appointment scheduling - Adapting patient questions during query submission - Prioritising patient queries based on clinical urgency - Signposting patients to the most appropriate care provider - Pre-programmed logic | [29] |
| Babylon GP at Hand | Includes an ‘AI’ triage system based on “chat-bot” symptom-checker. Patients are provided with advice, which might be self-care, message a clinician or have a telephone/video call. | - Two-way communication - Multiple choice questionnaires with optional free-text - Electronic health record integration - Prioritising patient queries based on clinical urgency - Signposting patients to the most appropriate care provide - Artificial intelligence method: unclear | [76] |
| Dr iQ | Algorithms with screening questions. Submissions are reviewed by a group of clinicians. | - Two-way communication - Multiple choice questionnaires only - No integration - Adapting patient questions during query submission - Pre-programmed logic | [39] |
| askmyGP | An online form detailing background and query, which is reviewed by administrative and clinician staff. | - One-way communication only - Multiple choice questionnaires with optional free-text - No integration - No artificial intelligence | [33,83,84] |
| MedicInfo | Free-text request sent to the GP. When answering, clinicians use one of four possible protocols. | - Two-way communication - Unstructured free-text only - No integration - No artificial intelligence | [64] |
| eConsulta | Users choose which health professional to direct their enquiry to and attach files, while keeping a record of previous interactions. | - Two-way communication - Unstructured free-text only - Electronic health record integration - No artificial intelligence | [26,31,33] |
| Digital Doctor Reception (DDR) | Patients complete a “medical history,” including cause of contact, background disease/s, and current inconvenience. Record form is handled digitally by a GP and continued through written digital dialogue. | - Two-way communication - Multiple choice questionnaires with optional free-text - Electronic health record integration - No artificial intelligence | [28] |
| Think Research platform and Novari platform | Patients can request visits to be completed through secure messaging, audio and/or video. Once a patient request is sent, the primary care provider receives a notification on the platform and through email. They log into the platform to see the request and respond through any of the three modalities. | - Two-way communication - Unstructured free-text only - No integration - No artificial intelligence | [25] |
| Epic’s MyChart patient portal | Patient chooses the e-visit condition that matches their symptoms and fills out a condition-specific questionnaire. Once the questionnaire is complete, the provider reviews it and responds to the patient with a treatment plan. | - Two-way communication - Multiple choice questionnaires only - Electronic health record integration - No artificial intelligence | [73] |
| Docly online consultation | Patients select their health problem, then fill in a guided questionnaire about their symptoms. Decision support algorithms recommend the most appropriate outcome to the clinician based on questionnaire answers, before either providing immediate advice or a further assessment is made by a GP. Health problems requiring more complex care are referred to specialist care services. Emergency situations are flagged by the smart algorithms, with patients receiving advice to contact urgent care services. | - Two-way communication - Multiple choice questionnaires only - No integration - Prioritising patient queries based on clinical urgency - Signposting patients to the most appropriate care provider - Pre-programmed logic | [43] |
| Zipnosis | Patients select from a list of chief complaints and complete a corresponding questionnaire regarding symptom types, duration, and severity, with an opportunity to provide any additional information to the primary care provider within a single free text entry at the end of the questionnaire. Providers receive virtual visit notifications via an app on their phone or computer and receive a summary of the patient’s chief complaint, symptoms, and free text comments. Along with the patient summary, the virtual visit software lists criteria based on national guidelines for bacterial sinusitis to help guide provider diagnosis. Based on the diagnoses chosen by the provider, the software will provide a drop-down menu of antibiotic choices for bacterial sinusitis or supportive care options for any diagnosis of sinusitis. | - Communication mode: Unclear - Multiple choice questionnaires with optional free-text - No integration | [96] |
| Unnamed (chat automation system) | Patients specify which medical problem they are seeking advice for and answer predefined questions derived for this problem, and then describe their symptoms using a chat with nurses and/or physicians. | - Two-way communication - Multiple choice questionnaires with optional free-text - No integration - Adapting patient questions during query submission - Artificial intelligence method: unclear | [45] |
| Unnamed (eVisit) | Patient submits answers to predetermined questions, which are reviewed by Advanced Practice Providers. | - Two-way communication - Multiple choice questionnaires only - No integration - Adapting patient questions during query submission - Pre-programmed logic | [35] |
| Unnamed (eVisit) | Patient selects their symptom/ healthcare concern from a menu and is directed to a symptom-specific structured set of algorithmic questions. Nurses review submissions. | - Two-way communication - Multiple choice questionnaires only - Electronic health record integration - Adapting patient questions during query submission - Pre-programmed logic | [79] |
| Unnamed (Online visits) | Using a branching logic program, medical history is obtained, along with patient-provided medication list, pharmacy of choice, and vital signs. Once the specific request is completed by the patient, it is sent to the patient’s physician for review. | - Two-way communication - Multiple choice questionnaires only - No integration - Adapting patient questions during query submission - Automated history taking - Pre-programmed logic | [99] |
| Unnamed (eVisits) | Patient-completed symptom specific question sets sent to a pool of Advanced Practice Providers via a secure online patient portal. | - Two-way communication - Multiple choice questionnaires only - No integration - No artificial intelligence | [34] |
| Unnamed (e-consultation) | Patients initiate an e-consultation by logging into a level 4 security portal, where a written message can be sent to the GP. | - Two-way communication - Unstructured free-text only - No integration - No artificial intelligence | [81] |
| Unnamed (Digital primary health care (DPHC) service) | Patients enter medical history, reason for contact, background disease(s), and current inconvenience. Record form is then digitally reviewed by a general practitioner (GP), who contacts the patient. | - Two-way communication - Multiple choice questionnaires with optional free-text - No integration - No artificial intelligence | [27] |
| Unnamed (chat-based and automated medical history–taking service) | Before the chat, an automated medical history–taking service is offered, where the patient responds to a questions about their chief complaint and current health status. | - Two-way communication - Multiple choice questionnaires with optional free-text - No integration - Adapting patient questions during query submission - Pre-programmed logic | [44] |
| Unnamed (e-visits) | Patients log into their secure personal health record internet portal and answer a series of questions about their condition. This written information is sent to the physicians, who make a diagnosis, order necessary care, put a note in the patients' electronic medical records, and reply to the patients via the secure portal within several hours. | - Two-way communication - Patient query format: unclear - No integration - No artificial intelligence | [98] |
| Unnamed (e-consultation) | An online text-based clinical consultation with the GP, used only for known health conditions. Accessed by secure login to the national health portal. Includes a medical assessment of the patient’s request and is considered complete when the doctor has processed the inquiry and provided an answer. | - Two-way communication - Patient query format: unclear - Electronic health record integration - No artificial intelligence | [46] |
| Unnamed (virtual visits) | Patients log in to an online portal to initiate a visit. Their primary care provider accepts the visit and responds in the most appropriate modality. The platform is primarily intended for two-way communication between the patient and the provider, however, patients can also request copies of lab results and prescription renewals. | - Two-way communication - Patient query format: unclear - No integration - No artificial intelligence | [32] |
| Unnamed (online consultations) | Allows patients to remotely and asynchronously contact a GP using a computer, smartphone, or tablet to ask questions and describe symptoms in writing. | - Communication mode: Unclear - Multiple choice questionnaires with optional free-text - No integration - No artificial intelligence | [47] |
| Unnamed (online consultations) | Allows patients to remotely and asynchronously contact a GP using a computer, smartphone, or tablet to ask questions and describe symptoms in writing. | - Communication mode: Unclear - Multiple choice questionnaires - No integration - No artificial intelligence | [47] |
| Unnamed (online consultations) | Allows patients to remotely and asynchronously contact a GP using a computer, smartphone, or tablet to ask questions and describe symptoms in writing. | - Communication mode: Unclear - Multiple choice questionnaires - No integration - Prioritising patient queries based on clinical urgency - Pre-programmed logic | [47] |
| Unnamed | Detailed description of features unavailable |  | [37,40-42,63,70,78,80,90,91,95,97,102,104,138,145  ] |

**References**

1. Atherton H, Brant H, Ziebland S, Bikker A, Campbell J, Gibson A, et al. The potential of alternatives to face-to-face consultation in general practice, and the impact on different patient groups: a mixed-methods case study. Health Serv Deliv Res 2018;6(20). PMID: 29889485. doi: 10.3310/hsdr06200.

2. Cowie J, Calveley E, Bowers G, Bowers J. Evaluation of a digital consultation and self-care advice tool in primary care: A multi-methods study. Int J Environ Res Public Health. 2018 02 May;15 (5) (896). doi: 10.3390/ijerph15050896.

3. NHS England. Prime Minister’s Challenge Fund: Improving Access to General Practice First Evaluation Report. NHS England; 2015 [updated October; cited 2020 1 Sept]; Available from: https://www.england.nhs.uk/wp-content/uploads/2015/10/pmcf-wv-one-eval-report.pdf.

4. Matheson C. Implementation of WebGP and Econsultations in Wessex GP Practices: Interim Update Report. Southampton, UK: Centre of Implementation Science; 2016 [updated June; cited 2020 1 Sept]; Available from: https://eprints.soton.ac.uk/397189/1/__soton.ac.uk_ude_personalfiles_users_cbm1a13_mydocuments_CBM%2520WebGP%2520update%252021062016IR.pdf.

5. Carter M, Fletcher E, Sansom A, Warren FC, Campbell JL. Feasibility, acceptability and effectiveness of an online alternative to face-to-face consultation in general practice: a mixed-methods study of webGP in six Devon practices. BMJ Open. 2018 May;8(2). PMID: WOS:000433129800107. doi: 10.1136/bmjopen-2017-018688.

6. Farr M, Banks J, Edwards HB, Northstone K, Bernard E, Salisbury C, et al. Implementing online consultations in primary care: a mixed-method evaluation extending normalisation process theory through service co-production. BMJ Open. 2018 Mar;8(3). PMID: WOS:000433881200172. doi: 10.1136/bmjopen-2017-019966.

7. Banks J, Farr M, Salisbury C, Bernard E, Northstone K, Edwards H, et al. Use of an electronic consultation system primary care: a qualitative interview study. Br J Gen Pract. 2018 Jan;68(666):E1-E8. PMID: WOS:000425962100001. doi: 10.3399/bjgp17X693509.

8. Edwards HB, Marques E, Hollingworth W, Horwood J, Farr M, Bernard E, et al. Use of a primary care online consultation system, by whom, when and why: Evaluation of a pilot observational study in 36 general practices in South West England. BMJ Open. 2017;7(11). doi: 10.1136/bmjopen-2017-016901.

9. Atherton H, Brant H, Ziebland S, Bikker A, Campbell J, Gibson A, et al. Alternatives to the face-to-face consultation in general practice: Focused ethnographic case study. Br J Gen Pract. 2018 April;68(669):e293-e300. doi: 10.3399/bjgp18X694853.

10. Casey M, Shaw S, Swinglehurst D. Experiences with online consultation systems in primary care: Case study of one early adopter site. Br J Gen Pract. 2017 November;67(664):e736-e43. doi: 10.3399/bjgp17X693137.

11. Jung C, Padman R. Virtualized healthcare delivery: Understanding users and their usage patterns of online medical consultations. Int J Med Inform. 2014;83(12):901-14. doi: 10.1016/j.ijmedinf.2014.08.004.

12. Padman R, Shevchik G, Paone S, Dolezal C, Cervenak J. eVisit: a pilot study of a new kind of healthcare delivery. Stud Health Technol Inform. 2010;160(Pt 1):262-6. PMID: 20841690.

13. Albert SM, Shevchik GJ, Paone S, Martich GD. Internet-based medical visit and diagnosis for common medical problems: experience of first user cohort. Telemed J E Health. 2011 May;17(4):304-8. PMID: 21457013. doi: 10.1089/tmj.2010.0156.

14. Jung C, Padman R, Shevchik G, Paone S. Who are portal users vs. early e-Visit adopters? A preliminary analysis. AMIA Annu Symp Proc. 2011:1070-9. PMID: 22195168.

15. Mehrotra A, Paone S, Martich GD, Albert SM, Shevchik GJ. Characteristics of Patients Who Seek Care via eVisits Instead of Office Visits. Telemed J E Health. 2013 2013/07/01;19(7):515-9. doi: 10.1089/tmj.2012.0221.

16. Peber E, Wästfelt E. Impact of digi-physical healthcare. [Master Thesis] Sweden: Lund University; 2020 [cited 2020 1 Sept]; Available from: https://lup.lub.lu.se/student-papers/search/publication/9015211.

17. Entezarjou A, Bolmsjö BB, Calling S, Midlöv P, Milos Nymberg V. Experiences of digital communication with automated patient interviews and asynchronous chat in Swedish primary care: a qualitative study. BMJ Open. 2020;10(7):e036585. doi: 10.1136/bmjopen-2019-036585.

18. Entezarjou A, Bonamy A-KE, Benjaminsson S, Herman P, Midlöv P. Human- Versus Machine Learning–Based Triage Using Digitalized Patient Histories in Primary Care: Comparative Study. JMIR Med Inform. 2020;8(9):e18930. PMID: 32880578. doi: 10.2196/18930.

19. Eldh AC, Sverker A, Bendtsen P, Nilsson E. Health Care Professionals' Experience of a Digital Tool for Patient Exchange, Anamnesis, and Triage in Primary Care: Qualitative Study. JMIR Hum Factors. 2020;7(4):e21698. PMID: 33315014. doi: 10.2196/21698.

20. North F, Crane SJ, Chaudhry R, Ebbert JO, Ytterberg K, Tulledge-Scheitel SM, et al. Impact of Patient Portal Secure Messages and Electronic Visits on Adult Primary Care Office Visits. Telemed J E Health. 2014 2014/03/01;20(3):192-8. doi: 10.1089/tmj.2013.0097.

21. North F, Crane SJ, Stroebel RJ, Cha SS, Edell ES, Tulledge-Scheitel SM. Patient-generated secure messages and eVisits on a patient portal: are patients at risk? J Am Med Inform Assoc. 2013;20(6):1143-9. doi: 10.1136/amiajnl-2012-001208.

22. Adamson SC, Bachman JW. Pilot Study of Providing Online Care in a Primary Care Setting. Mayo Clin Proc. 2010;85(8):704-10. doi: 10.4065/mcp.2010.0145.

23. Judson TJ, Odisho AY, Neinstein AB, Chao J, Williams A, Miller C, et al. Rapid design and implementation of an integrated patient self-triage and self-scheduling tool for COVID-19. J Am Med Inform Assoc. 2020;27(6):860-6. PMID: 32267928. doi: 10.1093/jamia/ocaa051.

24. Ipsos MORI, York Health Economics Consortium. Evaluation of Babylon GP at Hand: Final evaluation report. London2019 [cited 2020 1 Sept]; Available from: https://www.hammersmithfulhamccg.nhs.uk/media/156123/Evaluation-of-Babylon-GP-at-Hand-Final-Report.pdf.

25. Leung K, Qureshi S. Managing high frequency users of an electronic consultation system in primary care: a quality improvement project. BMJ Open Qual. 2021;10(2). PMID: WOS:000663456800001. doi: 10.1136/bmjoq-2020-001310.

26. Eccles A, Hopper M, Turk A, Atherton H. Patient use of an online triage platform: a mixed-methods retrospective exploration in UK primary care. Br J Gen Pract. 2019 May;69(682):E336-E44. PMID: WOS:000510826900006. doi: 10.3399/bjgp19X702197.

27. Nijland N, van Gemert-Pijnen J, Kelders SM, Brandenburg BJ, Seydel ER. Evaluation of the use of an "ask-the-expert" e-consultation service for support on health-related requests. Second International Conference on eHealth, Telemedicine, and Social Medicine; 10-16 Feb. 2010: IEEE; 2010. p. 72-6.

28. López Seguí F, Walsh S, Solans O, Adroher Mas C, Ferraro G, García-Altés A, et al. Teleconsultation Between Patients and Healthcare Professionals in the Catalan Primary Care Service: Message Annotation Analysis in a Retrospective Cross-Sectional Study. J Med Internet Res. 2020 Sep 17;22(9)(e19149). doi: 10.2196/19149.

29. López Seguí F, Vidal-Alaball J, Sagarra Castro M, García-Altés A, García Cuyàs F. General Practitioners’ Perceptions of Whether Teleconsultations Reduce the Number of Face-to-face Visits in the Catalan Public Primary Care System: Retrospective Cross-Sectional Study. J Med Internet Res. 2020 2020/3/16;22(3):e14478. doi: 10.2196/14478.

30. Fernández OS, Seguí FL, Vidal-Alaball J, Bonet Simo JM, Vian OH, Cabo PR, et al. Primary Care Doctor Characteristics That Determine the Use of Teleconsultations in the Catalan Public Health System: Retrospective Descriptive Cross-Sectional Study. JMIR Med Inform. 2020 2020/2/1;8(1):e16484. doi: 10.2196/16484.

31. Johansson A, Larsson M, Ivarsson B. General Practitioners' Experiences of Digital Written Patient Dialogues: A Pilot Study Using a Mixed Method. J Prim Care Community Health. 2020 Mar;11. PMID: WOS:000523514400001. doi: 10.1177/2150132720909656.

32. Stamenova V, Agarwal P, Kelley L, Fujioka J, Nguyen M, Phung M, et al. Uptake and patient and provider communication modality preferences of virtual visits in primary care: a retrospective cohort study in Canada. BMJ Open. 2020;10(7):e037064. doi: 10.1136/bmjopen-2020-037064.

33. Player M, O’Bryan E, Sederstrom E, Pinckney J, Diaz V. Electronic Visits For Common Acute Conditions: Evaluation Of A Recently Established Program. Health Aff. 2018 2018/12/01;37(12):2024-30. doi: 10.1377/hlthaff.2018.05122.

34. Nijhof D, Ingram A, Ochieng R, Roberts E-J, Poulton B, Ochieng B. Examining GP online consultation in a primary care setting in East Midlands, UK. BMC Health Serv Res. 2021 2021/09/30;21(1):1030. doi: 10.1186/s12913-021-07039-2.

35. Johnson KM, Dumkow LE, Burns KW, Yee MA, Egwuatu NE. Comparison of Diagnosis and Prescribing Practices Between Virtual Visits and Office Visits for Adults Diagnosed With Sinusitis Within a Primary Care Network. Open forum infectious diseases. 2019;6(9):ofz393-ofz. PMID: 31660415. doi: 10.1093/ofid/ofz393.

36. Cajander Å, Larusdottir M, Hedström G. The effects of automation of a patient-centric service in primary care on the work engagement and exhaustion of nurses. Qual User Exp. 2020 19 Sept;5(1):9. doi: 10.1007/s41233-020-00038-x.

37. Penza KS, Murray MA, Myers JF, Furst JW, Pecina JL. Management of Acute Sinusitis via e-Visit. Telemed J E Health. 2021. doi: 10.1089/tmj.2020.0047.

38. Penza KS, Murray MA, Pecina JL, Myers JF, Furst JW. Electronic Visits for Minor Acute Illnesses: Analysis of Patient Demographics, Prescription Rates, and Follow-Up Care Within an Asynchronous Text-Based Online Visit. Telemed J E Health. 2018 2018/03/01;24(3):210-5. doi: 10.1089/tmj.2017.0091.

39. Rohrer JE, Angstman KB, Adamson SC, Bernard ME, Bachman JW, Morgan ME. Impact of online primary care visits on standard costs: a pilot study. Popul Health Manag. 2010 Apr;13(2):59-63. PMID: 20415617. doi: 10.1089/pop.2009.0018.

40. Murray MA, Penza KS, Myers JF, Furst JW, Pecina JL. Comparison of eVisit Management of Urinary Symptoms and Urinary Tract Infections with Standard Care. Telemed J E Health. 2020 2020/05/01;26(5):639-44. doi: 10.1089/tmj.2019.0044.

41. Fagerlund AJ, Holm IM, Zanaboni P. General practitioners' perceptions towards the use of digital health services for citizens in primary care: A qualitative interview study. BMJ Open. 2019 01 May;9 (5)(e028251). doi: 10.1136/bmjopen-2018-028251.

42. Johansson A, Larsson M, Ivarsson B. Patients' Experiences With a Digital Primary Health Care Concept Using Written Dialogues: A Pilot Study. J Prim Care Community Health. 2020 01 Jan;11:2150132720910564. doi: 10.1177/2150132720910564.

43. Nilsson E, Sverker A, Bendtsen P, Eldh AC. A Human, Organization, and Technology Perspective on Patients’ Experiences of a Chat-Based and Automated Medical History–Taking Service in Primary Health Care: Interview Study Among Primary Care Patients. J Med Internet Res. 2021 2021/10/18;23(10):e29868. doi: 10.2196/29868.

44. Mehrotra A, Paone S, Martich GD, Albert SM, Shevchik GJ. A comparison of care at e-visits and physician office visits for sinusitis and urinary tract infection. JAMA Intern Med. 2013;173(1):72-4. PMID: 23403816. doi: 10.1001/2013.jamainternmed.305.

45. Zanaboni P, Fagerlund AJ. Patients' use and experiences with e-consultation and other digital health services with their general practitioner in Norway: Results from an online survey. BMJ Open. 2020 17 Jun;10 (6) (e034773). doi: 10.1136/bmjopen-2019-034773.

46. Kelley LT, Phung M, Stamenova V, Fujioka J, Agarwal P, Onabajo N, et al. Exploring how virtual primary care visits affect patient burden of treatment. Int J Med Inform. 2020 2020/09/01/;141:104228. doi: https://doi.org/10.1016/j.ijmedinf.2020.104228.

47. Turner A, Morris R, Rakhra D, Stevenson F, McDonagh L, Hamilton F, et al. Unintended consequences of online consultations: a qualitative study in UK primary care. Br J Gen Pract. 2022;72(715):e128. doi: 10.3399/BJGP.2021.0426.

48. Andersen KN, Nielsen JA, Kim S. Use, cost, and digital divide in online public health care: lessons from Denmark. Transforming Government- People Process and Policy. 2019 May;13(2):197-211. PMID: WOS:000484192600004. doi: 10.1108/tg-06-2018-0041.

49. NHS England, NHS Improvement. Online consultations research: Summary research findings. NHS England and NHS Improvement; 2019 [updated February; cited 2020 1 Sept]; Available from: https://www.england.nhs.uk/wp-content/uploads/2019/09/online-consultations-reserach-summary-of-findings.pdf.

50. Lawless M, Wright E, Davidson J. A collaborative approach to improving patient access in general practice: Impact of three different pilot schemes in 12 general practices in Greenwich. London J Prim Care. 2016;8(4):56-65. doi: 10.1080/17571472.2016.1173946.

51. McGrail KM, Ahuja MA, Leaver CA. Virtual Visits and Patient-Centered Care: Results of a Patient Survey and Observational Study. J Med Internet Res. 2017 2017/05/26;19(5):e177. doi: 10.2196/jmir.7374.

52. Peabody MR, Dai M, Turner K, Peterson LE, Mainous AG. Prevalence and Factors Associated with Family Physicians Providing E-Visits. J Am Board Fam Med. 2019;32(6):868. doi: 10.3122/jabfm.2019.06.190081.

53. Wilson G, Currie O, Bidwell S, Saeed B, Dowell A, Halim AA, et al. Empty waiting rooms: the New Zealand general practice experience with telehealth during the COVID-19 pandemic. N Z Med J. 2021 09 Jul;134(1537):89-101. PMID: 34239148.

54. Tarn DM, Hintz C, Mendez-Hernandez E, Sawlani SP, Bholat MA. Using virtual visits to care for primary care patients with COVID-19 symptoms. J Am Board Fam Med. 2021 February;34:S147-S51. doi: https://doi.org/10.3122/jabfm.2021.S1.200241.

55. Health Innovation Manchester. GM Digital First Primary Care: Patient and public insights: Workshop results. Report. Manchester: Health Innovation Manchester, 2021 Sept 2021. Report No.: 1 Contract No.: 1 Sept.

56. Bavafa H, Hitt LM, Terwiesch C. The Impact of E-Visits on Visit Frequencies and Patient Health: Evidence from Primary Care. Manage Sci. 2018;64(12):5461-80. PMID: 33033417. doi: 10.1287/mnsc.2017.2900.

57. Health Innovation Manchester. Greater Manchester digital primary care insight full report: Key findings from engagement June-July 2020. [Report] 2020 [cited 2021 1 Oct]; Available from: https://healthinnovationmanchester.com/wp-content/uploads/2020/12/PCIE-DPC-Full-report-FINAL-20.10.20-1.pdf.

58. Bertelsen P, Petersen L. Danish Citizens and General Practitioners' Use of ICT for their Mutual Communication. In: Sarkar I.N, Georgiou A, Mazzoncini de Azevedo Marques P, editors. MEDINFO 2015: eHealth-enabled Health; August; São Paulo, Brazil: IOS Press; 2015. p. 376-9.

59. Landgren S, Cajander Å. Non-use of Digital Health Consultations Among Swedish Elderly Living in the Countryside. Front Public Health. 2021 2021-September-10;9(1323). doi: 10.3389/fpubh.2021.588583.

60. Murphy M, Scott LJ, Salisbury C, Turner A, Scott A, Denholm R, et al. Implementation of remote consulting in UK primary care following the COVID-19 pandemic: a mixed-methods longitudinal study. Br J Gen Pract. 2021;71(704):e166-e77. doi: 10.3399/bjgp.2020.0948.

61. Hertzog R, Johnson J, Smith J, McStay FW, da Graca B, Haneke T, et al. Diagnostic Accuracy in Primary Care E-Visits: Evaluation of a Large Integrated Health Care Delivery System's Experience. Mayo Clin Proc. 2019;94(6):976-84. doi: 10.1016/j.mayocp.2019.02.011.

62. Bishop TF, Press MJ, Mendelsohn JL, Casalino LP. Electronic communication improves access, but barriers to its widespread adoption remain. Health Aff. 2013;32(8):1361-7. doi: 10.1377/hlthaff.2012.1151.

63. Ekman B, Thulesius H, Wilkens J, Lindgren A, Cronberg O, Arvidsson E. Utilization of digital primary care in Sweden: Descriptive analysis of claims data on demographics, socioeconomics, and diagnoses. Int J Med Inform. 2019;127:134-40. doi: 10.1016/j.ijmedinf.2019.04.016.
