# Supplementary material for: Understanding How the Design and Implementation of Online Consultations Affect Primary Care Quality: Systematic Review of Evidence With Recommendations for Designers, Providers, and Researchers
Source: J Med Internet Res. 2022 Oct 24;24(10):e37436. doi: 10.2196/37436 (PMC9621309; doi:10.2196/37436)
Supplement: Multimedia Appendix 6 [file jmir_v24i10e37436_app6.doc]

**Appendix 6: Quality appraisal of included studies using the Mixed Methods Appraisal Tool [54]**

| **Studies** |  | | **Question (see below for details)** | | | | | | | | | | | | | | | | | | | | | | | | |  |
| --- | --- | --- | --- | --- | --- | --- | --- | --- | --- | --- | --- | --- | --- | --- | --- | --- | --- | --- | --- | --- | --- | --- | --- | --- | --- | --- | --- | --- |
|  | **S1** | **S2** | **1.1** | **1.2** | **1.3** | **1.4** | **1.5** | **2.1** | **2.2** | **2.3** | **2.4** | **2.5** | **3.1** | **3.2** | **3.3** | **3.4** | **3.5** | **4.1** | **4.2** | **4.3** | **4.4** | **4.5** | **5.1** | **5.2** | **5.3** | **5.4** | **5.5** | **Overall score[[1]](#footnote-2)** |
| ADAMSON ET AL. [94] | Y | Y |  |  |  |  |  |  |  |  |  |  |  |  |  |  |  | ? | N | ? | Y | N |  |  |  |  |  | 1*/20% |
| ALBERT ET AL. [93] | Y | Y |  |  |  |  |  |  |  |  |  |  |  |  |  |  |  | Y | N | Y | Y | Y |  |  |  |  |  | 4****/80% |
| ANDERSEN ET AL. [70] | Y | Y |  |  |  |  |  |  |  |  |  |  |  |  |  |  |  | Y | Y | Y | Y | ? |  |  |  |  |  | 4****/80% |
| ATHERTON ET AL. [84] | Y | Y | Y | Y | Y | Y | Y |  |  |  |  |  |  |  |  |  |  |  |  |  |  |  |  |  |  |  |  | 5*****/100% |
| ATHERTON ET AL. [83] | Y | Y | Y | Y | Y | Y | Y |  |  |  |  |  |  |  |  |  |  | Y | N | Y | Y | Y | Y | Y | Y | Y | Y | 4****/80% |
| BANKS ET AL. [82] | Y | Y | Y | Y | Y | Y | Y |  |  |  |  |  |  |  |  |  |  |  |  |  |  |  |  |  |  |  |  | 5*****/100% |
| BAVAFA ET AL. [97] | Y | Y |  |  |  |  |  |  |  |  |  |  | Y | Y | N | N | ? |  |  |  |  |  |  |  |  |  |  | 2**/40% |
| BERTELSEN AND PETERSEN [102] | N | ? |  |  |  |  |  |  |  |  |  |  |  |  |  |  |  | N | Y | Y | N | N |  |  |  |  |  | 2**/40% |
| BISHOP ET AL. [80] | Y | Y | Y | N | Y | Y | Y |  |  |  |  |  |  |  |  |  |  |  |  |  |  |  |  |  |  |  |  | 4****/80% |
| CAJANDER ET AL. [45] | Y | Y | N | N | Y | Y | Y |  |  |  |  |  |  |  |  |  |  |  |  |  |  |  |  |  |  |  |  | 3***/60% |
| CARTER ET AL. [86] | Y | Y | Y | Y | Y | Y | Y |  |  |  |  |  |  |  |  |  |  | Y | N | Y | N | Y | Y | N | Y | Y | N | 3***/60% |
| CASEY ET AL. [69] | N | ? | N | N | Y | Y | N |  |  |  |  |  |  |  |  |  |  |  |  |  |  |  |  |  |  |  |  | 2**/40% |
| COWIE ET AL. [71] | Y | N | Y | N | Y | Y | Y |  |  |  |  |  |  |  |  |  |  | Y | N | N | N | Y | Y | Y | Y | Y | N | 2**/40% |
| ECCLES ET AL. [74] | Y | Y | N | N | Y | Y | Y |  |  |  |  |  |  |  |  |  |  | Y | Y | Y | N | Y | Y | Y | Y | Y | Y | 3***/60% |
| EDWARDS ET AL. [88] | Y | Y |  |  |  |  |  |  |  |  |  |  |  |  |  |  |  | Y | N | Y | Y | Y |  |  |  |  |  | 4****/80% |
| EKMAN ET AL. [104] | Y | Y |  |  |  |  |  |  |  |  |  |  |  |  |  |  |  | Y | Y | Y | Y | ? |  |  |  |  |  | 4****/80% |
| ELDH ET AL. [38] | Y | Y | Y | Y | Y | Y | Y |  |  |  |  |  |  |  |  |  |  |  |  |  |  |  |  |  |  |  |  | 5*****/100% |
| ENTEZARJOU ET AL. [30] | Y | Y | Y | Y | Y | Y | Y |  |  |  |  |  |  |  |  |  |  |  |  |  |  |  |  |  |  |  |  | 5*****/100% |
| ENTEZARJOU ET AL. [36] | Y | Y |  |  |  |  |  |  |  |  |  |  | N | Y | Y | Y | Y |  |  |  |  |  |  |  |  |  |  | 4****/80% |
| FAGERLUND ET AL. [81] | Y | Y | Y | Y | Y | Y | Y |  |  |  |  |  |  |  |  |  |  |  |  |  |  |  |  |  |  |  |  | 5*****/100% |
| FARR ET AL. [89] | Y | Y | Y | Y | Y | Y | Y |  |  |  |  |  |  |  |  |  |  | Y | N | Y | N | Y | N | Y | Y | Y | N | 3***/60% |
| FERNANDEZ ET AL. [26] | Y | Y |  |  |  |  |  |  |  |  |  |  | Y | Y | Y | N | ? |  |  |  |  |  |  |  |  |  |  | 3***/60% |
| HEALTH INNOVATION MANCHESTER [91] | N | ? | ? | N | Y | N | N |  |  |  |  |  |  |  |  |  |  |  |  |  |  |  |  |  |  |  |  | 1*/20% |
| HEALTH INNOVATION MANCHESTER [90] | N | ? | ? | ? | N | N | N |  |  |  |  |  |  |  |  |  |  | ? | Y | ? | ? | ? | N | ? | Y | N | ? | 0*/0% |
| HERTZOG ET AL. [138] | Y | Y |  |  |  |  |  |  |  |  |  |  | N | Y | Y | N | ? |  |  |  |  |  |  |  |  |  |  | 2**/40% |
| IPSOS MORI [76] | Y | Y | Y | Y | N | Y | Y |  |  |  |  |  |  |  |  |  |  | Y | Y | N | N | Y | N | Y | Y | Y | ? | 3***/60% |
| JOHANSSON ET AL. [27] | Y | Y |  |  |  |  |  |  |  |  |  |  |  |  |  |  |  | Y | N | Y | N | Y |  |  |  |  |  | 3***/60% |
| JOHANSSON ET AL. [28] | Y | Y | Y | Y | Y | Y | Y |  |  |  |  |  |  |  |  |  |  | N | N | Y | Y | Y | Y | Y | Y | Y | N | 3***/60% |
| JOHNSON ET AL. [96] | Y | Y |  |  |  |  |  |  |  |  |  |  | Y | Y | Y | N | ? |  |  |  |  |  |  |  |  |  |  | 3***/60% |
| JUDSON ET AL. [29] | N | ? |  |  |  |  |  |  |  |  |  |  |  |  |  |  |  | ? | ? | Y | Y | N |  |  |  |  |  | 2**/40% |
| JUNG AND PADMAN [75] | Y | Y |  |  |  |  |  |  |  |  |  |  | Y | Y | Y | Y | ? |  |  |  |  |  |  |  |  |  |  | 4****/80% |
| JUNG ET AL. [101] | N | ? |  |  |  |  |  |  |  |  |  |  | N | Y | Y | Y | ? |  |  |  |  |  |  |  |  |  |  | 3***/60% |
| KELLEY ET AL. [32] | Y | Y | Y | Y | Y | Y | Y |  |  |  |  |  |  |  |  |  |  | N | N | Y | N | Y | Y | Y | Y | Y | N | 2**/40% |
| LANDGREN AND CAJANDER [42] | Y | N | Y | N | Y | Y | N |  |  |  |  |  |  |  |  |  |  |  |  |  |  |  |  |  |  |  |  | 3***/60% |
| LAWLESS ET AL. [96] | Y | Y | Y | Y | ? | Y | ? |  |  |  |  |  |  |  |  |  |  | Y | N | Y | Y | Y | Y | Y | Y | N | Y | 3***/60% |
| LEUNG AND QURESHI [39] | Y | Y | Y | Y | N | Y | Y |  |  |  |  |  |  |  |  |  |  | ? | N | Y | Y | ? | Y | Y | Y | Y | Y | 2**/40% |
| MATHESON [87] | Y | N | Y | Y | Y | Y | Y |  |  |  |  |  |  |  |  |  |  | ? | N | N | Y | N | Y | Y | Y | Y | N | 1*/20% |
| MCGRAIL ET AL. [78] | Y | Y |  |  |  |  |  |  |  |  |  |  | Y | Y | Y | N | ? | Y | Y | Y | N | Y |  |  |  |  |  | 3***/60% |
| MEHROTRA ET AL. [103] | Y | Y |  |  |  |  |  |  |  |  |  |  | N | Y | Y | N | ? |  |  |  |  |  |  |  |  |  |  | 2**/40% |
| MEHROTRA ET AL. [98] | Y | Y |  |  |  |  |  |  |  |  |  |  |  |  |  |  |  | N | N | N | Y | Y |  |  |  |  |  | 2**/40% |
| MURPHY ET AL. [37] | Y | Y | Y | Y | Y | Y | Y |  |  |  |  |  |  |  |  |  |  | Y | N | Y | Y | Y | N | N | N | Y | N | 1*/20% |
| MURRAY ET AL. [34] | Y | Y |  |  |  |  |  |  |  |  |  |  |  |  |  |  |  | Y | N | Y | Y | Y |  |  |  |  |  | 4****/80% |
| NIJHOF ET AL. [43] | Y | N |  |  |  |  |  |  |  |  |  |  |  |  |  |  |  | Y | Y | N | Y | N |  |  |  |  |  | 3***/60% |
| NIJLAND ET AL. [64] | Y | Y |  |  |  |  |  |  |  |  |  |  |  |  |  |  |  | Y | ? | Y | ? | Y |  |  |  |  |  | 3***/60% |
| NILSSON ET AL. [44] | Y | Y | Y | Y | Y | Y | Y |  |  |  |  |  |  |  |  |  |  |  |  |  |  |  |  |  |  |  |  | 5*****/100% |
| NHS ENGLAND [125] | N | ? |  |  |  |  |  |  |  |  |  |  |  |  |  |  |  |  |  |  |  |  | Y | ? | ? | ? | ? | 1*/20% |
| NHS ENGLAND [63] | N | ? | ? | ? | Y | Y | ? |  |  |  |  |  |  |  |  |  |  | ? | ? | Y | ? | Y | ? | ? | Y | Y | ? | 2**/40% |
| NORTH ET AL. [100] | Y | Y |  |  |  |  |  |  |  |  |  |  | N | Y | Y | Y | ? |  |  |  |  |  |  |  |  |  |  | 3***/60% |
| NORTH ET AL. [72] | N | ? |  |  |  |  |  |  |  |  |  |  | N | Y | Y | Y | ? |  |  |  |  |  |  |  |  |  |  | 3***/60% |
| PADMAN ET AL. [92] | Y | Y | Y | ? | Y | Y | ? |  |  |  |  |  |  |  |  |  |  | Y | Y | Y | Y | Y | Y | Y | Y | Y | N | 3***/60% |
| PEABODY ET AL. [145] | Y | Y |  |  |  |  |  |  |  |  |  |  | Y | Y | Y | N | ? |  |  |  |  |  |  |  |  |  |  | 3***/60% |
| PEBER AND WÄSTFELT [65] | Y | Y |  |  |  |  |  |  |  |  |  |  |  |  |  |  |  | N | N | N | Y | N |  |  |  |  |  | 1*/20% |
| PLAYER ET AL. [73] | N | ? |  |  |  |  |  |  |  |  |  |  |  |  |  |  |  | ? | N | ? | Y | ? |  |  |  |  |  | 1*/20% |
| PENZA ET AL. [35] | Y | Y |  |  |  |  |  |  |  |  |  |  | Y | Y | Y | ? | ? |  |  |  |  |  |  |  |  |  |  | 3***/60% |
| PENZA ET AL. [79] | N | ? |  |  |  |  |  |  |  |  |  |  | N | Y | Y | N | ? |  |  |  |  |  |  |  |  |  |  | 2**/40% |
| ROHRER ET AL. [99] | Y | Y |  |  |  |  |  |  |  |  |  |  | N | Y | Y | Y | ? |  |  |  |  |  |  |  |  |  |  | 3***/60% |
| SEGUI ET AL. [31] | Y | Y |  |  |  |  |  |  |  |  |  |  |  |  |  |  |  | Y | N | Y | Y | Y |  |  |  |  |  | 4****/80% |
| SEGUI ET AL. [33] | Y | Y |  |  |  |  |  |  |  |  |  |  | N | Y | Y | N | Y | Y | N | Y | Y | Y |  |  |  |  |  | 3***/60% |
| STAMENOVA ET AL. [25] | Y | Y |  |  |  |  |  |  |  |  |  |  |  |  |  |  |  | N | Y | Y | N | ? |  |  |  |  |  | 2**/40% |
| TARN ET AL. [40] | Y | Y |  |  |  |  |  |  |  |  |  |  | N | Y | Y | Y | ? |  |  |  |  |  |  |  |  |  |  | 3***/60% |
| TURNER ET AL. [47] | Y | N | Y | N | Y | Y | N |  |  |  |  |  |  |  |  |  |  |  |  |  |  |  |  |  |  |  |  | 3***/60% |
| WILSON ET AL. [41] | Y | Y | Y | N | Y | Y | Y |  |  |  |  |  |  |  |  |  |  |  |  |  |  |  |  |  |  |  |  | 4****/80% |
| ZANABONI AND FAGERLUND [46] | Y | Y |  |  |  |  |  |  |  |  |  |  |  |  |  |  |  | Y | N | Y | ? | Y |  |  |  |  |  | 3***/60% |

**MMAT Questions [54]**

S1. Are there clear research questions?

S2. Do the collected data allow to address the research questions?

**1. Qualitative**

1.1. Is the qualitative approach appropriate to answer the research question?

1.2. Are the qualitative data collection methods adequate to address the research question?

1.3. Are the findings adequately derived from the data?

1.4. Is the interpretation of results sufficiently substantiated by data?

1.5. Is there coherence between qualitative data sources, collection, analysis and interpretation?

**2. Quantitative (randomized controlled trials)**

2.1. Is randomization appropriately performed?

2.2. Are the groups comparable at baseline?

2.3. Are there complete outcome data?

2.4. Are outcome assessors blinded to the intervention provided?

2.5 Did the participants adhere to the assigned intervention?

**3. Quantitative (nonrandomized)**

3.1. Are the participants representative of the target population?

3.2. Are measurements appropriate regarding both the outcome and intervention (or exposure)?

3.3. Are there complete outcome data?

3.4. Are the confounders accounted for in the design and analysis?

3.5. During the study period, is the intervention administered (or exposure occurred) as intended?

**4. Quantitative (descriptive)**

4.1. Is the sampling strategy relevant to address the research question?

4.2. Is the sample representative of the target population?

4.3. Are the measurements appropriate?

4.4. Is the risk of nonresponse bias low?

4.5. Is the statistical analysis appropriate to answer the research question?

**5. Mixed methods**

5.1. Is there an adequate rationale for using a mixed methods design to address the research question?

5.2. Are the different components of the study effectively integrated to answer the research question?

5.3. Are the outputs of the integration of qualitative and quantitative components adequately interpreted?

5.4. Are divergences and inconsistencies between quantitative and qualitative results adequately addressed?

5.5. Do the different components of the study adhere to the quality criteria of each tradition of the methods involved?

*Each section is marked out of 100 (20 marks for each question). For mixed methods studies, the overall quality score is the lowest score of the study components.*

1. Colour coding for MMAT to distinguish low to high ratings: Red (20%), grey (40%), yellow (60%), blue (80%), green (100%) [↑](#footnote-ref-2)
