# Supplementary material for: Understanding How the Design and Implementation of Online Consultations Affect Primary Care Quality: Systematic Review of Evidence With Recommendations for Designers, Providers, and Researchers
Source: J Med Internet Res. 2022 Oct 24;24(10):e37436. doi: 10.2196/37436 (PMC9621309; doi:10.2196/37436)
Supplement: Multimedia Appendix 7 [file jmir_v24i10e37436_app7.doc]

**Appendix 7: Low confidence findings for objective 1**

| **Theme** | **Subtheme** |
| --- | --- |
| **Effective** (providing care based on scientific knowledge to produce better clinical outcomes) | **Effectiveness:** Neutral-increased antibiotic prescribing rates (qualitative and quantitative)  **Description:** The same or higher rate of prescribed antibiotics than through traditional consultations  **CERQual rating:** Low  **CERQual explanation:** Low adequacy and low coherence  **References:** [34,65,98], n=3  **Exemplar data:**  *“Physicians were more likely to prescribe an antibiotic at an e-visit for either condition [sinusitis and UTI].”* [98] |
